# Production and Functional Verification of 8‐Gene (GGTA1, CMAH, β4GalNT2, hCD46, hCD55, hCD59, hTBM, hCD39)‐Edited Donor Pigs for Xenotransplantation

**DOI:** 10.1111/cpr.70028

**Published:** 2025-04-06

**Authors:** Jiaoxiang Wang, Kaixiang Xu, Tao Liu, Heng Zhao, Muhammad Ameen Jamal, Gen Chen, Xiaoying Huo, Chang Yang, Deling Jiao, Taiyun Wei, Hanfei Huang, Hongfang Zhao, Jianxiong Guo, Fengchong Wang, Xiong Zhang, Kai Liu, Siming Qu, Gang Wang, Hui Guo, Gang Chen, Hong‐Ye Zhao, Zhong Zeng, Kefeng Dou, Hong‐Jiang Wei

**Affiliations:** ^1^ Yunnan Province Key Laboratory for Porcine Gene Editing and Xenotransplantation, Yunnan Agricultural University Kunming China; ^2^ Yunnan Province Xenotransplantation Research Engineering Center, Yunnan Agricultural University Kunming China; ^3^ Faculty of Animal Science and Technology, Yunnan Agricultural University Kunming China; ^4^ College of Veterinary Medicine, Yunnan Agricultural University Kunming China; ^5^ First Affiliated Hospital of Kunming Medical University Kunming China; ^6^ Guizhou Provincial People's Hospital Guiyang China; ^7^ Department of Pharmacology Innovative Institute of Basic Medical Sciences of Zhejiang University Hangzhou China; ^8^ Institute of Organ Transplantation Tongji Hospital, Tongji Medical College, Huazhong University of Science and Technology Wuhan China; ^9^ Department of Hepatobiliary Surgery Xijing Hospital, Air Force Medical University Xi'an China

**Keywords:** complement regulatory protein, kidney xenotransplantation, pig, thrombomodulin, xenoantigens

## Abstract

Gene‐edited (GE) pig‐to‐human xenotransplantation continues to make breakthroughs, but which kind of gene combination is suitable for organ‐specific transplantation remains unclear. In this study, we utilised CRISPR/Cas9 gene editing technology, PiggyBac transposon system, and serial somatic cell cloning technology to develop GTKO/CMAHKO/β4GalNT2KO/hCD46/hCD55/hCD59/hCD39/hTBM 8 gene‐edited cloned (GEC) donor pigs and performed pig‐to‐non‐human primate (NHP) transplantation to evaluate the effectiveness of these GEC pigs. The 8‐GEC pigs were obtained by recloning with a 33‐day‐old 8‐GEC fetus with O blood type, which was generated after cell transfection, screening of cell colonies, and somatic cell cloning. Molecular identification at DNA, mRNA, and protein levels confirmed successful 8‐gene editing. Three copies of transgenes were identified by droplet digital polymerase chain reaction and whole genome sequencing, which were inserted into the introns of pig RFTN1 and MYO10 genes, as well as the intergenic region between PRLR and LOC110257300 genes of these 8‐GEC pigs. The 8‐GEC pigs also exhibited the ability of germline transmission when mated with our previously generated 4‐GEC male pigs. Moreover, antigen–antibody binding assay and complement‐dependent cytotoxicity assay demonstrated that 8‐gene editing effectively reduced the immune incompatibility and kidney xenograft from 8‐GEC pigs survived for 15 and 17 days in two NHPs, respectively. Postoperatively, the recipient serum antibodies IgA, IgG and IgM, complements C3 and C4, coagulation indicators PT, APTT, TT and FIB, as well as most electrolytes and liver function indicators remained relatively stable. Serum creatinine was normal within 10 days post operation. However, the kidney xenograft developed active antibody‐mediated rejection at necropsy, characterised by the deposition of antibodies IgG and IgM, as well as complements C4d, C3c and C5b‐C9, infiltration of CD68^+^ macrophages, and micro‐thrombotic embolism of glomerular capillaries, etc. In conclusion, we successfully developed fertile 8‐GEC pigs, which effectively alleviated immune rejection and exerted life‐supporting kidney function in the recipients.

## Graphical abstract

Schematic representation of generation of 8‐gene‐edited cloned pigs and their verification by pig to non‐human primate kidney xenotransplantation.

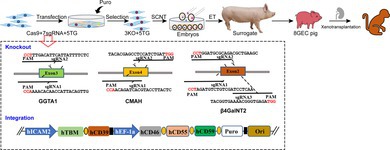

## 1 Introduction

Xenotransplantation is an effective way to solve the global donor shortage. With the rapid development of gene editing technology and new immunosuppressants, the United States of America (USA) has taken a leading step in conducting subclinical research on gene‐edited (GE) pig‐to‐human xenotransplantation. In 2022, the world's first GE pig heart xenograft was transplanted into a human body at the University of Maryland (UAM) Medical Center in the United States of America (USA), which survived for 60 days [1]. In 2023, the heart xenografts from GE pigs were transplanted in 2 brain‐dead patients for 66 h [2], and one live patient who survived for 42 days [3]. In 2023, a kidney graft from a GE pig to a brain‐dead patient was transplanted at New York University Langone Medical Center in the USA, which functioned normally for 61 days [4]. Additionally, seven cases of multi‐GE pig kidneys have been successfully transplanted into human brain‐dead subjects with variable survival times (54 h (2 cases) [5], 74 h [6], 7 days [7], 12 days (2 cases) [8], 32 days [9]). While, in 2024, researchers also transplanted a kidney from a GE pig into a live patient who survived for 56 days, thus establishing a new record for functional GE pig kidneys in humans [10]. In recent years, pig‐to‐human xenotransplantation has made new breakthroughs and is soon expected to enter the clinical phase.

The major barriers in pig‐to‐human organ transplantation are immunological incompatibility, physiological dysfunction, and biosafety. To overcome these barriers, multiple GE donor pigs with different gene combinations are constructed, and xenografts from these GE pigs have been transplanted to NHPs, and even to brain‐dead and live humans, which demonstrated their effectiveness in the long‐term survival of xenografts. In pig‐to‐NHP xenotransplantation, the transplantation of a pig heart with 3‐GE (GTKO/hCD46/hTBM) into baboons functionally survived up to 195 days [11], while that of 10 GE (GTKO/CMAHKO/β4GalNT2KO/GHRKO/hCD46/hCD55/hTBM/hEPCR/hCD47/hHO‐1) survived for 225 days [12]. The kidneys from 2‐GE (GTKO/hCD55) pigs functionally survived up to 499 days in rhesus monkeys [13]. Recently, the transplantation of 11 gene‐edited pig kidneys into cynomolgus monkeys extended the survival up to 758 days [14]. Furthermore, two cases of recent heart transplants and multiple cases of kidney transplantation from GE pigs to human patients with organ failure or brain‐dead humans exhibited the feasibility of donor pigs to save human lives [1, 2]. Collectively, multiple genetic modifications are necessary to overcome xenotransplantation barriers. However, which kind of genetic combination is optimal for organ‐specific xenotransplantation remains undetermined.

The glycogen α‐Gal, Neu5Gc, and Sda synthesised by three proteins GGTA1, CMAH, and β4GalNT2, respectively, are known xenoantigens. Once the pig xenograft is transplanted into the recipient, the corresponding natural antibodies bind to these carbohydrate antigens on the surface of porcine cells and induce hyperacute rejection (HAR) [15]. Therefore, inactivation of these xenoantigens, that is, triple knockout (TKO) is necessary. Besides these, there may be multiple unknown xenoantigens in pigs [16]. After antigen presentation and B cell activation, corresponding antibodies may still be produced, thereby activating the complement system to attack porcine cells. Therefore, expression of human complement‐regulatory proteins (CD46, CD55, CD59) is necessary for inhibiting complement system activation. Furthermore, the development of thrombotic microangiopathy becomes increasingly evident with long‐term graft survival [17]. Thus, addition of the coagulation regulatory factor, that is, thrombomodulin (TBM) can effectively improve these immune responses [18, 19]. In addition, activated immune cells and injured cells can secrete adenosine triphosphate (ATP). Ectonucleoside triphosphate diphosphohydrolase‐1, also referred to as CD39, can promote immunosuppression and protect the tissue against inflammation and thrombosis via cleaving ATP to adenosine monophosphate [20].

Previously, we successfully generated 4 GE pigs (GTKO/hCD55/hTBM/hCD39) for xenotransplantation and found that kidney xenografts from 4 GE pigs survived for 11 days in NHP [21]. Based on our experience, in this study, we used CRISPR‐Cas9, PiggyBac transposon and somatic cell nuclear transfer (SCNT) technology to construct 8‐GE (GTKO/CMAHKO/ β4GalNT2KO/hCD46/hCD55/hCD59/hTBM/hCD39) *Diannan* miniature pigs and performed kidney transplantation from pig to rhesus monkey to evaluate the effectiveness of these GE donor pigs.

## 2 Materials and Methods

Experimental animals used in this study and all surgical procedures were approved by the Animal Ethics Committee of Yunnan Agricultural University.

### 2.1 Vector Construction

To target porcine GGTA1, CMAH and β4GalNT2, 2–3 sgRNAs were designed at the 3rd exon, 4th exon, and 2nd exon of these three genes, respectively. All the sgRNAs were expressed using the U6 promoter and subcloned into the PX458 vector (Addgene ID: 112220) to construct the PX458‐sgRNAs targeting vector. Meanwhile, the PB‐hICAM2‐hTBM‐P2A‐hCD39‐hEF‐1α‐hCD46‐P2A‐hCD55‐P2A‐hCD59‐P2A‐Puro vector driven by the human ICAM2 promoter (hTBM and hCD39) and the human EF‐1α promoter (hCD46, hCD55 and hCD59) was constructed and confirmed by sequencing, followed by plasmid extraction and cell transfection. All transgenes were cDNA except CD46, which was the whole sequence.

### 2.2 Cell Transfection, Screening and Identification

The *Diannan* miniature pig fetal fibroblasts (PFFs) with O blood type were cultured in DMEM containing 10% fetal bovine serum (FBS) at 38°C and 5% CO_2_. Once the cells attained 60% confluence, the cells were harvested and counted. Approximately 3 × 10^5^ cells were suspended in the electro‐transfection buffer containing PX458‐sgRNA plasmid (8 μg), PB‐hICAM2‐hTBM‐P2A‐hCD39‐hEF‐1α‐hCD46‐P2A‐hCD55‐P2A‐hCD59‐P2A‐Puro plasmid (8 μg) and transposase plasmid (4 μg) were incubated for 5 min, and electroporated under the program of EH‐100 by a Lonza 4D‐nucleofector X Unit (EH‐100, Germany). After electroporation, the cells were plated into T25 flasks for 24 h in DMEM supplemented with 10% FBS. Then, 3 μg/mL puromycin was added to the culture medium for 24–48 h to select successfully transfected cells. Subsequently, the surviving cells were digested and ~80 cells were seeded in a 100 mm diameter dish and cultured for 8 days to obtain single‐cell colonies, which were further harvested for genotyping by PCR and Sanger sequencing using the primers shown in Table S1. Cells with biallelic knockout (KO) of GGTA1, CMAH and β4GalNT2, and hICAM2‐hTBM‐P2A‐hCD39‐hEF‐1α‐hCD46‐P2A‐hCD55‐P2A‐hCD59‐Puro integrated colonies were selected as donor cells for SCNT.

### 2.3 Somatic Cell Nuclear Transfer and Embryo Transfer

Oocyte collection, in vitro maturation, SCNT, and embryo transfer were performed as described previously [22]. Briefly, cultured cumulus‐oocyte complexes were isolated from cumulus cells by treating them with 0.1% (w/v) hyaluronidase. The oocytes with the first polar body were selected and cultured in porcine zygote medium‐3 (PZM‐3) media containing 10% FBS, 0.0171 g/mL sucrose, and 0.01 μg/mL colchicine, and incubated at 38°C in a 5% CO_2_ incubator for 0.5~1 h. The first polar body was enucleated via gentle aspiration using a bevelled pipette in TLH‐PVA, while the donor cells were injected into the perivitelline space of the enucleated oocytes. The reconstructed embryos were fused with a single direct current pulse of 25 V/mm for 20 μs using the Electro Cell Fusion Generator (LF201, NEPA GENE Co. Ltd. Japan) in fusion medium. Embryos were then cultured in PZM‐3 for 0.5–1 h and activated with a single pulse of 150 V/mm for 100 μs in activation medium. The embryos were equilibrated in PZM‐3 supplemented with 5 μg/mL cytochalasin B for 2 h at 38.5°C in a humidified atmosphere with 5% CO_2_, 5% O_2_ and 90% N_2_ (APM‐30D, ASTEC, Japan) and then cultured in PZM‐3 medium with the same culture conditions described above until embryo transfer. The SCNT embryos were surgically transferred into the oviducts of the recipients, and a viable fetus was obtained through caesarean section after 33 days of pregnancy. A fetal fibroblast cell line was established and subjected to PCR, T7EI enzyme digestion experiment, Sanger sequencing, flow cytometry, immunofluorescence, and WB identification. Foetuses with biallelic TKO and targeted overexpression of 5 genes were selected as donor cells for recloning. After the completion of the gestation period (~114 days), GEC piglets were obtained through natural delivery and identified by the same method of fetus identification.

### 2.4 Genotype Identification of Cell Colonies, Cloned Foetuses and Piglets

The screened cell colonies were directly lysed, and the whole‐genome DNA from cell colonies, fetal tissue, and cloned piglet ear tissues was extracted using a high‐salt method and used as a template for the amplification of the target gene fragment through polymerase chain reaction (PCR). For cell colonies, we first confirmed the integration of five genes through gel electrophoresis imaging and then used Sanger sequencing analysis to determine the genotypes of the target regions of three genes (GGTA1, CMAH, and β4GalNT2), and finally, the cells with KO of these genes (TKO) along with the successful integration of five genes, were selected as a donor for cloning. The 8‐GE status of foetuses and cloned piglets was analysed by PCR and Sanger sequencing.

### 2.5 Karyotyping

Karyotyping was performed in the Kunming Institute of Zoology, Chinese Academy of Sciences, China. Briefly, PFFs at 70%~80% confluence were incubated with 0.2 μg/mL colchicine (Colcemide‐Gibco etc.) for 2 h, and treated with hypotonic solution Na‐Citrate/NaCl at 37°C for 15 min. Subsequently, cells were fixed with methanol/acetic acid glacial (3:1) for 20 min at −20°C and washed twice with methanol/acetic as stated above, and Giemsa stained according to manufacturer guidelines and analysed for metaphase chromosome smear analysis using SmartType.

### 2.6 Droplet Digital Polymerase Chain Reaction (ddPCR) for Transgenic Copy Numbers

The samples including the human blood (positive control), the pre‐transfected porcine cells (negative control), the drug‐selected cell lines (C12#), the fetal cells (C12F01) and the heart, liver, lung, kidney, spleen and other tissues of the cloned pig derived from foetuses were collected and extracted DNA. Then, these DNA samples were digested by MseI (Cat#FD0984, ThermoFisher), EagI (Cat#FD0334, ThermoFisher) and AsisI (Cat#FD2094, ThermoFisher) restriction endonuclease at 37°C for 2 h and inactivated at 65°C and 85°C for 20 min. The digested product was diluted to 5 ng/μL and used as a ddPCR template. The primers and probes corresponding to hCD46, hCD55, hCD59, hTBM, hCD39 and GAPDH genes (Table S1), ddPCR super mix (Cat#1863024, Bio‐Rad), and DNA template were mixed to prepare a 25 uL reaction system. Then, ddPCR droplets were generated with QX100 Droplet Generator (Bio‐Rad) and transferred to a 96 well plate. PCR reactions were performed at 94°C for 5 min, 94°C for 30 s, 56°C for 1 min (40 cycle) and 98°C for 10 min. After the reaction, the 96 well plate was placed in Bio‐Rad Droplet Reader, and QuantaSoft Software was used to set up the experimental design and read the experiment. Once the program was finished, the copy numbers were analysed through gating, according to the manufacturer's instructions (Bio‐Rad).

### 2.7 Quantitative Polymerase Chain Reaction (qPCR)

To evaluate the mRNA expression levels of hCD46, hCD55, hCD59, hTBM and hCD39, total RNA from heart, liver, lung, kidney and other tissues of WT (*n* = 3) and GEC pigs (*n* = 3), as well as human umbilical cord tissues, was extracted using TRIzol reagent (Cat#ET111‐01‐V2, TransGen Biotech) according to the manufacturer's instructions, and the concentration and RNA quality were detected. Complementary DNA (cDNA) was synthesised from total RNA using a PrimeScript RT reagent Kit (Cat#RR047B, TaKaRa) and was used as a template to perform qPCR in a TB green‐based qPCR instrument (CFX‐96, Bio‐Rad, USA). The reaction was performed in a 20 μL reaction mixture comprising 10 μL of 2 × TB Green Premix Ex Taq (Tli RNaseH Plus) (Cat#RR420A, TaKaRa), 1 μL of cDNA, 1 μL of forward primer, 1 μL of reverse primer and 7 μL of ddH_2_O (primers listed in Table S1). The reaction program is as follows: 95°C for 30 s, followed by 40 cycles of 95°C for 10 s and 62°C for 45 s. Three technical replicates were conducted for each sample, and the relative expression levels of target genes were quantified by 2^−ΔΔct^.

### 2.8 Flow Cytometry

The cells were collected when confluence reached 70%–80%, digested with trypsin, and centrifuged at 1200 rpm for 3 min. Then cells were resuspended with 50 μL PBS and incubated with primary antibodies (Table S2) according to 1 × 10^3^ cells/μL at 37°C in the dark for 15–30 min. After that, cells were washed with PBS and incubated with corresponding secondary antibodies at 37°C in the dark for 30 min. Cells were washed with PBS and detected by using a CytoFLEX flow cytometer (Beckman Coulter, USA). Data were analysed by FlowJo‐V10.8.1 software.

### 2.9 Immunofluorescence

The paraffin‐embedded tissue blocks were cut into 5 μm, transferred to glass slides, dewaxed using xylene and gradient alcohol, retrieved antigens with EDTA buffer (Cat#G1207, Servicebio Bio) at 92°C–98°C for 15 min, and cooled at room temperature. Then, sections were washed with PBS three times (each time 3 min), incubated with autofluorescence quencher A (Cat#G1221, Servicebio Bio) at RT in the dark for 15 min, washed with PBS three times again, incubated with FBS at RT for 30 min, and dried. The dried sections were incubated with corresponding antibodies (Table S2). For visualisation, corresponding secondary antibodies (Table S2) were diluted with PBS containing 10% FBS (v/v = 1:200) and used to incubate sections at 4°C in the dark for 2 h, and a negative control was incubated with PBS containing 10% FBS. Then, sections were washed with PBS three times and stained with DAPI (Cat#G1012, Servicebio Bio) for 3 min. After washing with PBS for 1 min, autofluorescence quencher B (Cat#G1221, Servicebio Bio) was added for 5 min and washed three times again. Finally, sections were mounted with anti‐fluorescence quencher (Cat#G1401, Servicebio Bio) and imaged using an OLYMPUS BX53 fluorescence microscope, and the fluorescence intensity of different samples was compared using ImageJ software.

### 2.10 Whole Genome Sequencing (WGS)

The control (34dDN201618F06) was derived from WT fetal tissue, which was used for cell line establishment to perform 8 gene editing. The samples 8TGC12F01P01, 8TGC12F01P08 and 8TGC12F01P17 were from 8‐GEC porcine ear tissues, while the samples 8TGC12F01P13, 8TGC12F01P22 and 8TGC12F01P23 were from 8‐GEC porcine kidney, kidney and liver tissues, respectively. These samples were used for DNA extraction. A total of 0.2 μg of DNA sample was used as input material for DNA library preparation. Briefly, the genomic DNA was fragmented to approximately 350 bp using a Covaris LE220R‐plus (Covaris, USA). The DNA fragments were then end‐polished, A‐tailed, and ligated with full‐length adapters for Illumina sequencing, followed by PCR amplification [23]. The PCR products were purified using the AMPure XP system (Beckman Coulter, Beverly, USA). Library quality was assessed on the Agilent 5400 system (AATI) and quantified by real‐time PCR (1.5 nM). After library quality control, different libraries were pooled based on effective concentration and targeted data amount. The 5′ end of each library was phosphorylated and cyclized. Subsequently, loop amplification was performed to generate DNA nanoballs [24]. These DNA nanoballs were then loaded onto the flow cell of the DNBSEQ‐T7 for sequencing at BioHuaxing Bioinformatics Technology Co. Ltd. (Beijing, China). In total, deep sequencing of samples was conducted with a depth of > 50× per individual. Subsequently, quality control (QC) analysis was conducted by using the fastp software [25] with default parameters to remove low‐quality reads and ensure the reliability of downstream analysis.

### 2.11 Identification of Insertion Location of Transgenes

To elucidate the integration site of an insert sequence with 15,086 bp, quality‐controlled sequencing reads were aligned to this insert sequence utilising the bwa‐mem2 [26], followed by processing with samtools software [27] for generating the alignment file in BAM format. From these BAM files, we extracted reads that exhibit alignment to regions flanking both sides of the insert sequence. Given the premise that upon integration into the host genome, reads encompassing the junctions between the insert and genomic DNA will contain sequences alignable to both the insert and the porcine reference genome (GCF_000003025.6_Sscrofa11.1), these flanking reads were subjected to a secondary alignment against the genome (GCF_000003025.6_Sscrofa11.1) using the same methodology. The insertion locus was subsequently inferred based on the results of both alignments.

To construct a new reference genome that includes the integrated insert sequence, the insertion site was identified and the insert sequence was concatenated with the genomic sequence at this precise location. Following the above method, all sequencing reads were aligned to this new composite reference genome. Subsequently, for detailed characterisation of the integration event, the depth information of a specific region encompassing the insert sequence along with 20 kb of upstream and downstream flanking sequences was extracted and was performed on this region using a sliding window approach (window = 100 bp). The resulting data were then visualised with Python for the number of copies of the inserted fragment.

### 2.12 Off‐Target Analysis by WGS

For off‐target effect validation, the offline version of Cas‐OFFinder software [28] was utilised to predict potential off‐target sites within the pig genome (GCF_000003025.6_Sscrofa11.1). The prediction was based on the NGG protospacer adjacent motif (PAM) and allowed for a maximum of 4 base mismatches. Following the identification of candidate off‐target loci, we proceeded with treatment‐control variant calling using the Mutect2 tool from GATK [27]. This analysis compared the treatment group against the control group to detect variants potentially induced by the CRISPR/Cas9 system. Subsequently, the variant profiles of 8‐GE samples were aggregated and analysed to identify recurrent variants. Variants that occurred at a higher frequency across multiple samples were highlighted as candidates for further investigation, indicating their potential significance as true off‐target effects or other relevant genomic alterations.

### 2.13 Pig‐Monkey Cross‐Matching Experiment

Pig PBMCs and rhesus monkey serum were used for cross‐matching to select recipient monkeys with low antibody titers and low complement‐dependent toxicity for kidney transplantation.

Whole blood (10 mL each) of healthy rhesus monkeys was collected, and serum was separated. At the same time, 10 mL of whole blood from wild‐type pigs and gene‐edited donor pigs was collected to isolate PBMCs. Based on the antigen–antibody binding assay, inactivated serum from each monkey was incubated with wild‐type pig and gene‐edited donor pig PBMCs, and then treated with anti‐IgG antibody (Cat#628411, Invitrogen) and anti‐IgM antibody (Cat#A18842, Invitrogen), respectively, and the levels of binding of IgG and IgM to pig cells were detected by cell flow cytometry, and monkeys with lower MFI were selected as xenograft recipients.

For complement‐dependent cytotoxicity experiment, 50 uL of 50% inactivated monkey serum was incubated with pig PBMC cells for 2 h. The reaction was then terminated using 0.5 mL pre‐cooled PBS, samples were centrifuged at 300 × g for 3 min, the supernatant was discarded, and incubated with 50 uL rabbit complement serum (1: 10, Cat#S7764, Sigma) at room temperature for 30 min. Finally, cells were stained with PI staining solution (Cat#GA1174, Servicebio Bio) for 10 min, and analysed for cell death using a CytoFLEX flow cytometer (Beckman Coulter, USA).

### 2.14 Pig‐To‐Monkey Kidney Xenotransplantation and Postoperative Care

We carried out pig‐to‐nonhuman primate kidney transplantation at Yunnan Province Key Laboratory for Porcine Gene Editing and Xenotransplantation, Yunnan Agricultural University, Kunming. On the day of surgery, both kidneys of the recipient monkey were removed, an 8‐GE pig kidney was transplanted, and a gastrostomy was performed to facilitate postoperative medication. After the operation, urine biochemistry, blood biochemistry, immune indicators, coagulation function, infection, immunosuppressant blood drug concentration, etc. were tested regularly. The blood flow of the transplanted kidney was detected by ultrasound. Clinical, and medical staff and veterinary professionals provided postoperative care, including various tests, indicators and the mental state of the recipient monkey, and applied corresponding treatment methods.

### 2.15 H&E Staining

Tissue samples were fixed in 4% paraformaldehyde for 48–72 h, processed by an automatic tissue processor (Yd‐12p, Jinhua Yidi, China) and embedded in a paraffin block (Yd‐6D, Jinhua Yidi, China). The paraffin blocks were cut into 3‐μm‐thick sections using a Microm HM 325 microtome (Thermo Scientific, USA) and allowed to dry on glass slides overnight at 37°C. Thereafter, the tissue sections were deparaffinised in xylene and rehydrated through graded ethanol dilutions. Sections were stained with haematoxylin–eosin (H&E) (Cat#G1120, Solarbio) according to the manufacturer's instructions. Imaged using an OLYMPUS BX53 fluorescence microscope and analysed using the software of accessories.

### 2.16 Immunohistochemical Analysis of Xenografts

The paraffin‐embedded tissue blocks were cut into 3 μm, transferred to glass slides, dewaxed using xylene and gradient alcohol, put into a microwave oven to retrieve antigens with EDTA buffer (Cat#G1207, Servicebio Bio) at 92°C–98°C for 15 min, and cooled at room temperature. Then, sections were washed with PBS three times (each time 3 min), incubated in 3% H_2_O_2_ solution for 15 min, washed with PBS three times again, incubated with FBS at RT for 30 min, and dried. The dried sections were incubated with corresponding antibodies (CD57, 1:200; CD68, 1:200, Table S2; Table S2) at 4°C overnight. After washing with PBS three times, sections were incubated with 5 μg/mL HRP‐conjugated goat anti‐rabbit/mouse IgG antibody for 20 min. After washing three times again, sections were stained with fresh DAB (KIT‐9901, Elivision TM plus Polyer HRP IHC Kit, China) solution in the dark for 1 min. PBS wash 3 min × 3. Haematoxylin counter‐staining, and neutral gum sealing slides. Imaged using an OLYMPUS BX53 fluorescence microscope and analysed using software of accessories.

### 2.17 Statistical Analysis

All data were analysed using SPSS 22.0 software package (IBM Crop, Armonk, NY) and expressed as mean ± standard deviation of the mean (SD). Statistical significance was defined as *p < 0.05, **p < 0.001.

## 3 Results

### 3.1 Generation of the 8‐GEC Pigs

**FIGURE 1:**
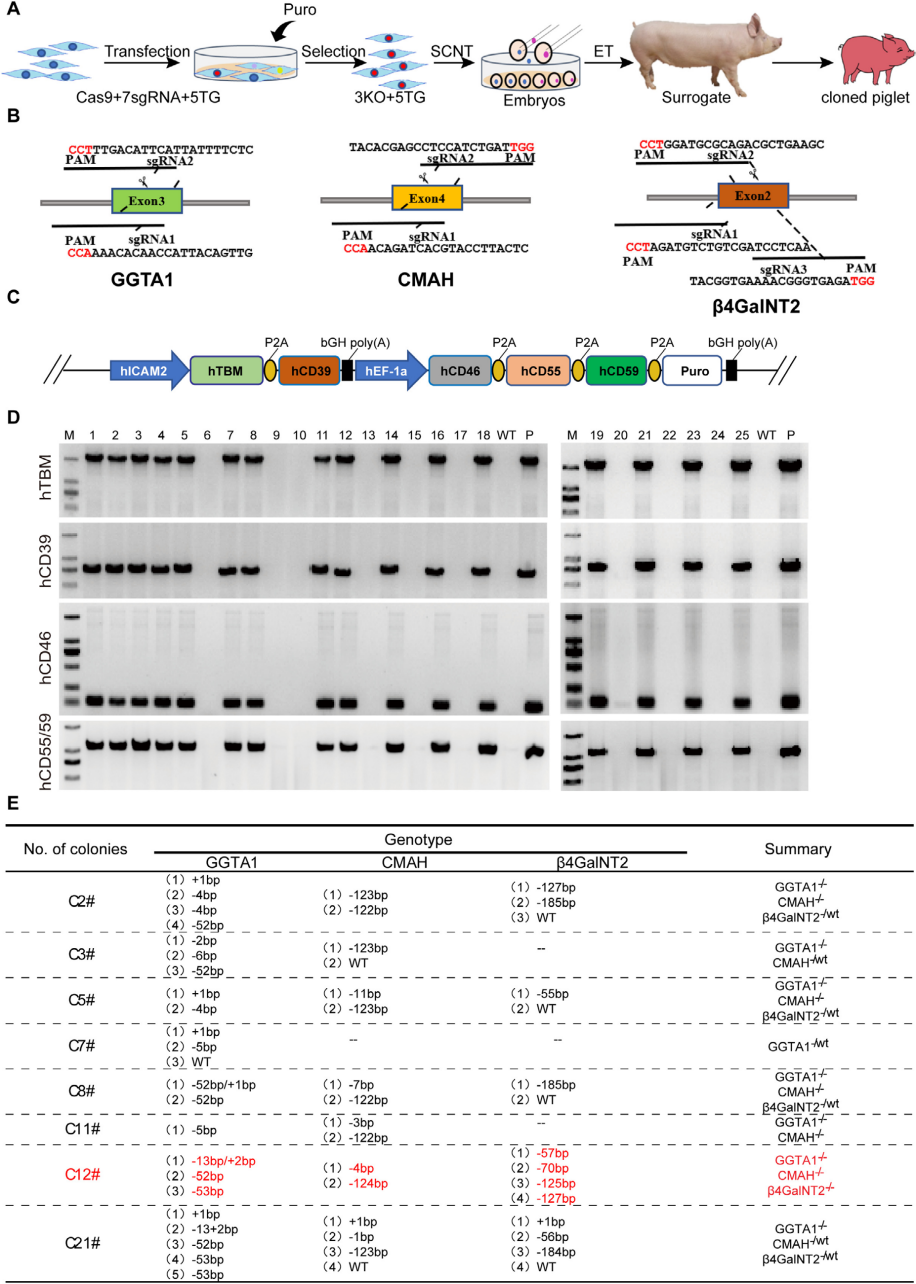
Construction of gene editing vectors for TKO, with 5 genes overexpression and screening out cell colonies. (A) Schematic diagram for the generation of 8‐GE cloned piglets. (B) Knockout of porcine GGTA1, CMAH, β4GalNT2 gene by CRISPR/Cas9 targeting Exon 3, 4 and 2, respectively. (C) The expression of human CD46, hCD55 and hCD59 genes was driven by human EF‐1α promoter, and the expression of hTBM and hCD39 gene were driven by human ICAM2 promoter. (D) hTBM, hCD39, hCD46, hCD55 and hCD59 genes were successfully intergrated into pig genome in cell colonies by PCR identification. A total of 16 cell colonies were positive for transgene integration. (E) The sequence of the targeting region of GGTA1, CMAH, β4GalNT2 genes in cell colonies by Sanger sequencing after transfection and drug selection. Only the C12# colony was biallelic TKO along with integration of 5 human genes. Abbreviations: TKO = triple knockout of GGTA1, CMAH, β4GalNT2 genes.

**FIGURE 2:**
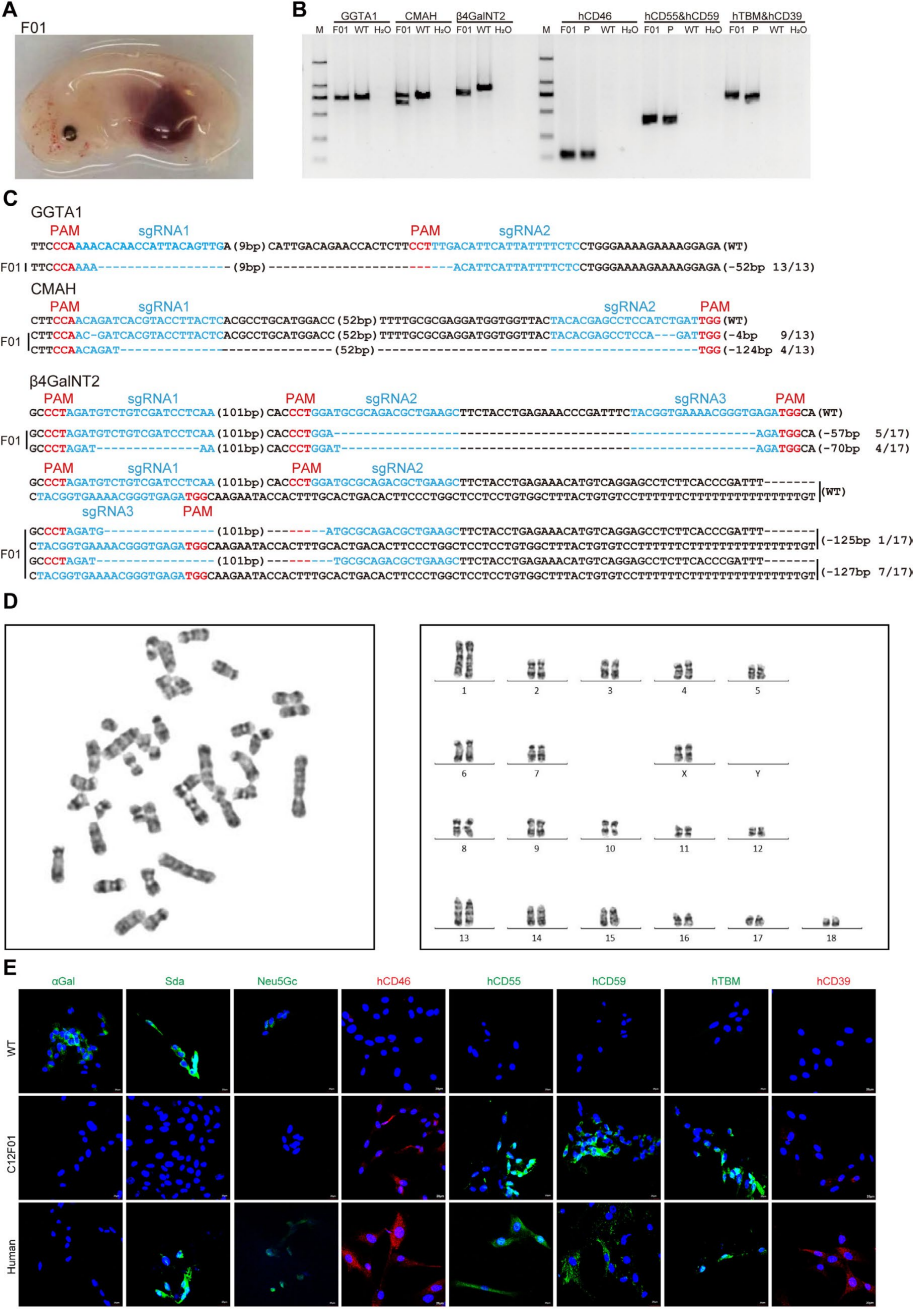
Generation and identification of gene editing cloned foetuses. (A) Photo of cloned fetus (C12F01) obtained after 33‐day pregnancy. (B) The biallelic TKO and integration of 5 genes into fetal genome was confirmed by PCR. (C) The sequence of the targeting region of GGTA1, CMAH, and β4GalNT2 genes in fetus by Sanger sequencing. (D) Karyotyping analysis of cloned fetus. (E) The protein expression of αGal, Neu5Gc, Sda, hCD46, hCD55, hCD59, hTBM and hCD39 genes in cloned fetal fibroblasts was confirmed by immunofluorescence. Abbreviations: TKO = triple knockout of GGTA1, CMAH, β4GalNT2 genes.

**FIGURE 3:**
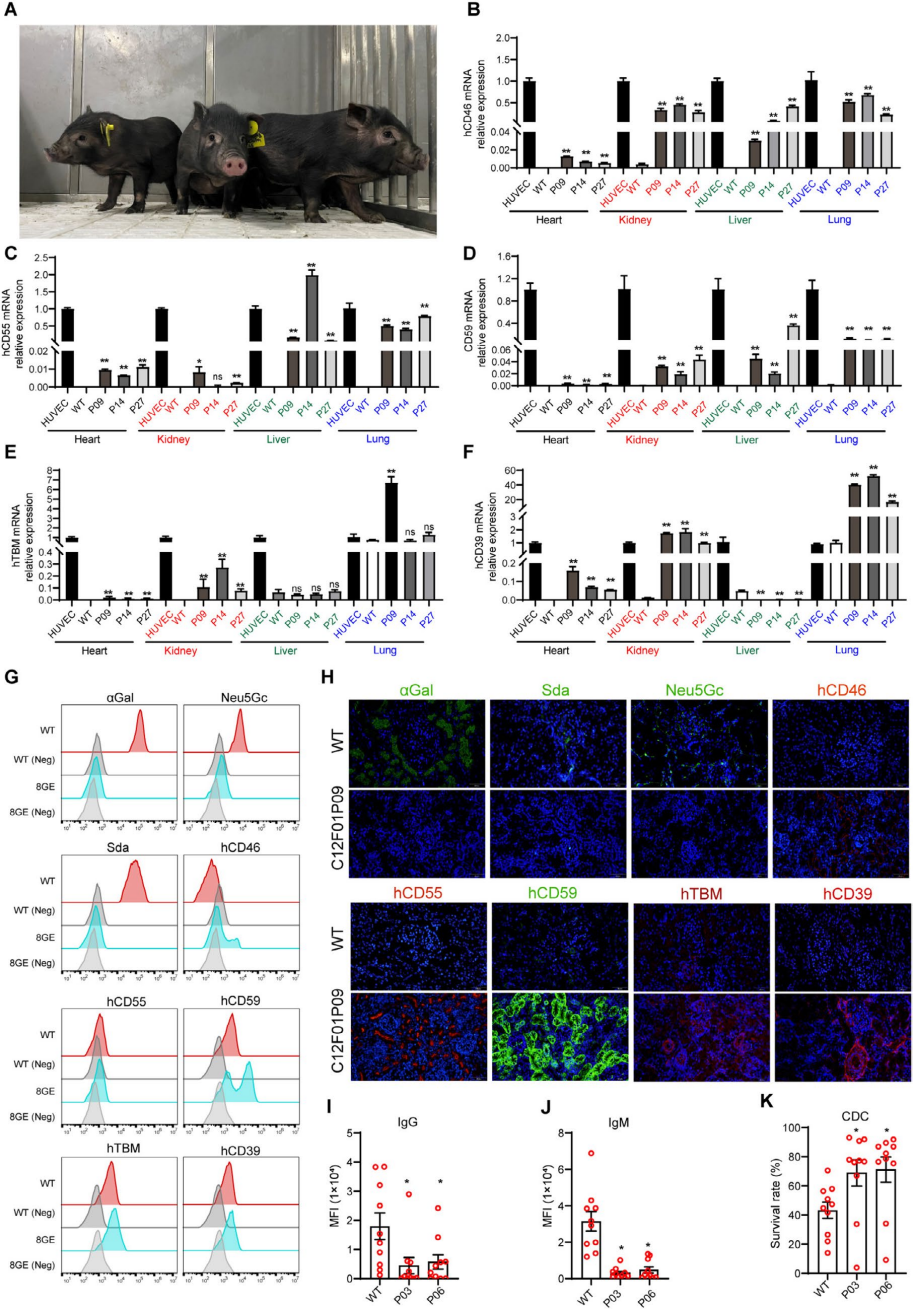
Phenotype of 8‐GE cloned pigs and crossmatch with rhesus monkeys. (A) Photo of 8‐GEC piglets. (B–F) The mRNA expression levels of hCD46, hCD55, hCD59, hTBM, and hCD39 genes in heart, kidney, liver, and lung tissues of GEC pigs (*n* = 3; P09, P14, P27). WT, (*n* = 3); HUVEC, Human umbilical vein endothelial cell. (G) The expression of α αGal, Neu5Gc, Sda, hCD46, hCD55, hCD59, hTBM and hCD39 in GEC pig aortic endothelial cells was confirmed by flow cytometry. (H) The expression of αGal, Sda, Neu5Gc,hCD46, hCD55, hCD59, hTBM and hCD39 in GEC pig kidney was confirmed by immunofluorescence. (I) The levels of monkey IgG binding to 8‐GE porcine PBMCs. (J) The levels of monkey IgM binding to 8‐GE porcine PBMCs. (K) The survival rate of 8‐GEC porcine PBMCs after incubation with each monkey serum. Abbreviations: GE = gene edited; GEC = gene edited cloned; PBMCs = peripheral blood mononuclear cells; WT = wild type.

To produce the GTKO/CMAHKO/β4GalNT2KO/hCD46/hCD55/hCD59/hTBM/hCD39 8‐GEC donor pigs, the CRISPR/Cas9 system, the PiggyBac transposons system, and somatic cell cloning were applied, and the constructed plasmids were co‐transfected into fetal fibroblasts of *Diannan* miniature pigs. After drug selection and genotyping, 8‐GE cell colonies were obtained and used as donors for somatic cell cloning to obtain 8‐GEC pigs (Figure 1A). We designed two sgRNAs targeting the 3rd exon of the GGTA1 gene, two sgRNAs targeting the 4th exon of the CMAH gene, and three sgRNAs targeting the 2nd exon of β4GalNT2 genes to simultaneously conduct TKO in the *Diannan* miniature pigs (Figure 1B). The overexpression of hCD46, hCD55, hCD59, hTBM and hCD39 genes was achieved using the human ICAM‐2 promoter (hTBM and hCD39) and the human EF‐1α promoter (hCD46, hCD55 and hCD59) (Figure 1C). After electroporation and drug selection, a total of 25 cell colonies were obtained. At first, these colonies were identified by PCR, and 64% (16/25) carried human genes (Figure 1D). Then, 8 colonies were randomly selected for Sanger sequencing, and results showed that 7 colonies (C2#, C3#, C5#, C8#, C11#, C12# and C21#) for GGTA1, 5 colonies (C2#, C5#, C8#, C11# and C12#) for CMAH and 1 colony (C12#) for β4GalNT2 were biallelic KO. Therefore, among the 25 colonies, only the C12# colony was biallelic TKO along with the integration of 5 human genes (Figure 1E, Table 1); thus, it was used as a donor for SCNT, and reconstructed embryos were transferred into 4 surrogate sows. One of them became pregnant, and a 33‐day‐old viable fetus (C12F01) was obtained (Figure 2A, Table 2), which also carried 5 human genes along with biallelic TKO as verified by genotyping (Figure 2B,C) and exhibited a normal karyotype (Figure 2D). Moreover, immunofluorescence analysis also showed that three xenoantigens αGal, Neu5Gc and Sda were deficient, and hCD46, hCD55, hCD59, hTBM and hCD39 genes were expressed in the fetus (Figure 2E). Therefore, we used the fetal fibroblasts as donor cells for re‐cloning and transferred them into 17 surrogate sows. Six of them became pregnant, giving birth to a total of 28 piglets, of which 24 survived, with an average of 1.4 live pigs per sow (Table 3). Out of the surviving piglets, 17 grew up healthy and reached sexual maturity (Figure 3A, Table 4). Their genotype of GGTA1, CMAH and β4GalNT2 was consistent with the 8GEC fetus. Among them, the GGTA1 gene has a 52 bp deletion mutation, the CMAH gene has 4 and 124 bp deletion mutations, and the β4GalNT2 gene has 57, 70, 125 and 127 bp deletion mutations (Table 5). To confirm whether the CRISPR‐Cas9 system caused off‐targets in 8‐GEC pigs, we detected 1024 potential gRNA off‐target sites on a whole genome scale (Table S3). The result of off‐target analysis showed minimal off‐target mutations in the 8‐GEC porcine genome. There are mutations in the intergenic region of chromosomes 1 and 14, as well as in the LOC110255214 gene on chromosome 12, also named β‐1,4 N‐acetylgalactosaminyltransferase 2‐like, which is similar to the β4GalNT2 gene (Table S4).

**TABLE 1:** Genotyping of colonies by Sanger sequencing.

Gene	No. of colonies	Sequence	Mutation
GGTA1	WT	TCCTTTTCTTTTCCCAGGAGAAAATAATGAATGTCAAAGGAAGAGTGGTTCTGTCAATGCTGCTTGTCTCAACTGTAATGGTTGTGTTTT	
	C2#	TCCTTTTCTTTTCCCAGGAGAAAATAATGAATGT‐‐‐‐‐‐‐‐‐‐‐‐‐‐‐‐‐‐‐‐‐‐‐‐‐‐‐‐‐‐‐‐‐‐‐‐‐‐‐‐‐‐‐‐‐‐‐‐TTTT TCCTTTTCTTTTCCCAGGAGAAAATAATGAATGaTCAAAGGAAGAGTGGTTCTGTCAATGCTGCTTGTCTCAACTGTAATGGTTGTGTTTT TCCTTTTCTTTTCCCAGGAGAAAATAATGA‐‐‐‐CAAAGGAAGAGTGGTTCTGTCAATGCTGCTTGTCTCAACTGTAATGGTTGTGTTTT	−52 bp 1/4 +1 bp 2/4 −4 bp 1/4
	C3#	TCCTTTTCTTTTCCCAGGAGAAAATAATGAATGT‐‐‐‐‐‐‐‐‐‐‐‐‐‐‐‐‐‐‐‐‐‐‐‐‐‐‐‐‐‐‐‐‐‐‐‐‐‐‐‐‐‐‐‐‐‐‐‐‐‐‐‐TTTT TCCTTTTCTTTTCCCAGGAGAAAATAATG‐‐‐‐‐‐AAAGGAAGAGTGGTTCTGTCAATGCTGCTTGTCTCAACTGTAATGGTTGTGTTTT TCCTTTTCTTTTCCCAGGAGAAAATAATGAA‐‐TCAAAGGAAGAGTGGTTCTGTCAATGCTGCTTGTCTCAACTGTAATGGTTGTGTTTT	−52 bp 1/5 −6 bp 3/5 −2 bp 1/5
	C5#	TCCTTTTCTTTTCCCAGGAGAAAATAATGAA‐‐‐‐AAAGGAAGAGTGGTTCTGTCAATGCTGCTTGTCTCAACTGTAATGGTTGTGTTTT TCCTTTTCTTTTCCCAGGAGAAAATAATGAATGATCAAAGGAAGAGTGGTTCTGTCAATGCTGCTTGTCTCAACTGTAATGGTTGTGTTTT	−4 bp 6/8 +1 bp 2/8
	C7#	TCCTTTTCTTTTCCCAGGAGAAAATAATG‐‐‐‐‐CAAAGGAAGAGTGGTTCTGTCAATGCTGCTTGTCTCAACTGTAATGGTTGTGTTTT TCCTTTTCTTTTCCCAGGAGAAAATAATGAATGATCAAAGGAAGAGTGGTTCTGTCAATGCTGCTTGTCTCAACTGTAATGGTTGTGTTTT TCCTTTTCTTTTCCCAGGAGAAAATAATGAATGTCAAAGGAAGAGTGGTTCTGTCAATGCTGCTTGTCTCAACTGTAATGGTTGTGTTTT	−5 bp 2/6 +1 bp 2/6 WT 2/6
	C8#	TCCTTTTCTTTTCCCAGGAGAAAATAATGAATGT‐‐‐‐‐‐‐‐‐‐‐‐‐‐‐‐‐‐‐‐‐‐‐‐‐‐‐‐‐‐‐‐‐‐‐‐‐‐‐‐‐‐‐‐‐‐‐‐‐‐‐‐TTTT TCCTTTTCTTTTCCCAGGAGAAAATAATGAATGT‐‐‐‐‐‐‐‐‐‐‐‐‐‐‐‐‐‐‐‐‐‐‐‐‐‐‐‐‐‐‐‐‐‐‐‐‐‐‐‐‐‐‐‐‐‐‐‐‐‐‐TTTTT	−52 bp 5/11 −51 bp/+1 bp 6/11
	C11#	TCCTTTTCTTTTCCCAGGAGAAAATAATG‐‐‐‐‐CAAAGGAAGAGTGGTTCTGTCAATGCTGCTTGTCTCAACTGTAATGGTTGTGTTTT	−5 bp 2/2
	C12#	TCC‐‐‐‐‐‐‐‐‐‐‐‐‐‐‐‐‐‐‐‐‐‐‐‐‐‐‐‐‐‐‐‐‐‐‐‐‐‐‐‐‐‐‐‐‐‐‐‐‐‐‐‐‐ATGCTGCTTGTCTCAACTGTAATGGTTGTGTTT TCCTTTTCTTTTCCCAGG‐‐‐‐‐‐‐‐‐‐‐‐GTTGTCAAAGGAAGAGTGGTTCTGTCAATGCTGCTTGTCTCAACTGTAATGGTTGTGTTT TCCTTTTCTTTTCCCAGGAGAAAATAATGAATGT‐‐‐‐‐‐‐‐‐‐‐‐‐‐‐‐‐‐‐‐‐‐‐‐‐‐‐‐‐‐‐‐‐‐‐‐‐‐‐‐‐‐‐‐‐‐‐‐‐‐‐‐TTTT	−53 bp 6/8 −12 bp /+2 bp 1/8 −52 bp 1/8
	C21#	TCC‐‐‐‐‐‐‐‐‐‐‐‐‐‐‐‐‐‐‐‐‐‐‐‐‐‐‐‐‐‐‐‐‐‐‐‐‐‐‐‐‐‐‐‐‐‐‐‐‐‐‐‐‐ATGCTGCTTGTCTCAACTGTAATGGTTGTGTTT TCCTTTTCTTTTCCCAGG‐‐‐‐‐‐‐‐‐‐‐‐GTTGTCAAAGGAAGAGTGGTTCTGTCAATGCTGCTTGTCTCAACTGTAATGGTTGTGTTT TCCTTTTCTTTTCCCAGGAGAAAATAATGAATGT‐‐‐‐‐‐‐‐‐‐‐‐‐‐‐‐‐‐‐‐‐‐‐‐‐‐‐‐‐‐‐‐‐‐‐‐‐‐‐‐‐‐‐‐‐‐‐‐‐‐‐‐TTTT TCCTTTTCTTTTCCCAGGAGAAAATAATGAATGTCAAAGGAAGAGTGGTTCTGTCAATGCTGCTTGTCTCAACTGTAATGGTTGTGTTT TCCTTTTCTTTTCCCAGG‐‐‐‐‐‐‐‐‐‐‐‐TGTTCAAAGGAAGAGTGGTTCTGTCAATGCTGCTTGTCTCAACTGTAATGGTTGTGTTT	−53 bp 4/9 −12 bp/+2 bp 2/9 −52 bp 1/9 WT 1/9 +1 bp 1/9
CMAH	WT	AACAGATCACGTACCTTACTC‐60 bp‐CCTGCTTTTGCGCGAGGATGGTGGTTACTACACGAGCCTCCATCTGATTGGCTG	
	C2#	AACAG‐‐‐‐‐‐‐‐‐‐‐‐60 bp‐‐‐‐‐‐‐‐‐‐‐‐‐‐‐‐‐‐‐‐‐‐‐‐‐‐‐‐‐‐‐‐‐‐‐‐‐‐‐‐‐‐‐‐‐‐‐‐‐‐TGATTGGCTG AACA‐‐‐‐‐‐‐‐‐‐‐‐‐60 bp‐‐‐‐‐‐‐‐‐‐‐‐‐‐‐‐‐‐‐‐‐‐‐‐‐‐‐‐‐‐‐‐‐‐‐‐‐‐‐‐‐‐‐‐‐‐‐‐‐‐TGATTGGCTG	−122 bp 1/6 −123 bp 5/6
	C3#	AACA‐‐‐‐‐‐‐‐‐‐‐‐‐60 bp‐‐‐‐‐‐‐‐‐‐‐‐‐‐‐‐‐‐‐‐‐‐‐‐‐‐‐‐‐‐‐‐‐‐‐‐‐‐‐‐‐‐‐‐‐‐‐‐‐‐TGATTGGCTG AACAGATCACGTACCTTACTC‐60 bp‐CCTGCTTTTGCGCGAGGATGGTGGTTACTACACGAGCCTCCATCTGATTGGCTG	−123 bp 3/8 WT 5/8
	C5#	AACA‐‐‐‐‐‐‐‐‐‐‐‐‐60 bp‐‐‐‐‐‐‐‐‐‐‐‐‐‐‐‐‐‐‐‐‐‐‐‐‐‐‐‐‐‐‐‐‐‐‐‐‐‐‐‐‐‐‐‐‐‐‐‐‐‐TGATTGGCTG AACA‐‐‐‐‐‐‐‐ACCTTACTC‐60 bp‐CCTGCTTTTGCGCGAGGATGGTGGTTACTACACGAGCCTCCATCTGATTGGCTG AACAGATCACGTACCTTACTC‐60 bp‐CCTGCTTTTGCGCGAGGATGGTGGTTACTACACGAGCCTCCATCTGATTGGCTG	−123 bp 3/5 −8 bp 1/5 WT 1/5
	C8#	AACA‐‐‐‐‐CGTACCTTACTC‐60 bp‐CCTGCTTTTGCGCGAGGATGGTGGTTACTACACGAGCCTCCAT‐‐GATTGGCTG AACAG‐‐‐‐‐‐‐‐‐‐‐‐60 bp‐‐‐‐‐‐‐‐‐‐‐‐‐‐‐‐‐‐‐‐‐‐‐‐‐‐‐‐‐‐‐‐‐‐‐‐‐‐‐‐‐‐‐‐‐‐‐‐‐‐TGATTGGCTG	−7 bp 3/8 −122 bp 5/8
	C11#	AACA‐‐‐‐‐‐‐‐‐‐‐‐‐60 bp‐‐‐‐‐‐‐‐‐‐‐‐‐‐‐‐‐‐‐‐‐‐‐‐‐‐‐‐‐‐‐‐‐‐‐‐‐‐‐‐‐‐‐‐‐‐‐‐‐‐TGATTGGCTG AAC‐GATCACGTACCTTACTC‐60 bp‐CCTGCTTTTGCGCGAGGATGGTGGTTACTACACGAGCCTCCAT‐‐GATTGGCTG	−123 bp 2/3 −3 bp 1/3
	C12#	AAC‐GATCACGTACCTTACTC‐60 bp‐CCTGCTTTTGCGCGAGGATGGTGGTTACTACACGAGCCTCCA‐‐‐GATTGGCTG AACAGAT‐‐‐‐‐‐‐‐‐‐‐‐‐‐60 bp‐‐‐‐‐‐‐‐‐‐‐‐‐‐‐‐‐‐‐‐‐‐‐‐‐‐‐‐‐‐‐‐‐‐‐‐‐‐‐‐‐‐‐‐‐‐‐‐‐‐TGGCTG	−4 bp 7/9 −124 bp 2/9
	C21#	AACA‐‐‐‐‐‐‐‐‐‐‐‐‐60 bp‐‐‐‐‐‐‐‐‐‐‐‐‐‐‐‐‐‐‐‐‐‐‐‐‐‐‐‐‐‐‐‐‐‐‐‐‐‐‐‐‐‐‐‐‐‐‐‐‐‐TGATTGGCTG AAC‐GATCACGTACCTTACTC‐60 bp‐CCTGCTTTTGCGCGAGGATGGTGGTTACTACACGAGCCTCCATTGGATTGGCTG AACAAGATCACGTACCTTACTC‐60 bp‐CCTGCTTTTGCGCGAGGATGGTGGTTACTACACGAGCCTCCATTGGATTGGCTG AACAGATCACGTACCTTACTC‐60 bp‐CCTGCTTTTGCGCGAGGATGGTGGTTACTACACGAGCCTCCATTGGATTGGCTG	−123 bp 1/8 −1 bp 2/8 +1 bp 2/8 WT 3/8
β4GalNT2	WT	GATGTCTGTCGATCCTCAA‐68 bp‐TTCAGTCTCCTCAACTCACCCATCCCGTCCCCCACCCTGGATGCG−48 bp‐GTGAGA	
	C2#	GA‐‐‐‐‐‐‐‐‐‐‐‐‐‐‐‐68 bp‐‐‐‐‐‐‐‐‐‐‐‐‐‐‐‐‐‐‐‐‐‐‐‐‐‐‐‐‐‐‐‐‐‐‐‐‐‐‐‐‐‐‐‐TGCG‐48 bp‐GTGAGA GATGTCTGTCGATCCTCAA‐68 bp‐TTCAGTCTCCTCAACTCACCCATCCCGTCCCCCACCCTGGATGCG‐48 bp‐GTGAGA	−185 bp 3/7 WT 4/7
	C8#	GATGTCTGTCGATCCTCAA‐68 bp‐TTCAGTCTCCTCAACTCACCCATCCCGTCCCCCACCCTGGATGCG‐48 bp‐GTGAGA GA‐‐‐‐‐‐‐‐‐‐‐‐‐‐‐‐68 bp‐‐‐‐‐‐‐‐‐—‐‐‐‐‐‐‐‐‐‐‐‐‐‐‐‐‐‐‐—‐‐‐‐‐‐‐‐‐‐TGCG‐48 bp‐GTGAGA	WT 2/8 −185 bp 6/8
	C12#	GAT‐‐‐‐‐‐‐‐‐‐‐‐‐‐‐‐68 bp‐‐‐‐‐‐‐‐‐‐‐‐‐‐‐‐‐‐‐‐‐‐‐‐‐‐‐‐‐‐‐‐‐‐‐‐‐‐‐‐‐‐‐TGCG‐48 bp‐GTGAGA GATG‐‐‐‐‐‐‐‐‐‐‐‐‐‐‐68 bp‐‐‐‐‐‐‐‐—‐‐‐‐‐‐‐‐‐‐‐‐‐‐‐‐‐‐‐‐—‐‐‐‐‐‐‐‐ATGCG‐48 bp‐GTGAGA GAT‐‐‐‐‐‐‐‐‐‐‐‐‐‐AA‐68 bp‐TTCAGTCTCCTCAACTCACCCATCCCGTCCCCCACCCTGGAT‐‐‐‐47 bp‐‐‐‐‐AGA GATGTCTGTCGATCCTCAA‐68 bp‐TTCAGTCTCCTCAACTCACCCATCCCGTCCCCCACCCTGGA—‐‐47 bp‐‐‐‐‐AGA	−127 bp 6/14 −125 bp 2/14 −70 bp 3/14 −57 bp 3/14
	C21#	GATGTCTGTCGATCCTCAA‐68 bp‐TTCAGTCTCCTCAACTCACCCATCCCGTCCCCCACCCTGGATGCG‐48 bp‐GTGAGA GA—‐‐‐‐‐‐‐‐‐‐‐‐‐68 bp‐‐‐‐—‐‐‐‐‐‐‐‐‐‐‐‐‐‐‐‐‐‐‐‐‐‐—‐‐‐‐‐‐‐‐‐‐‐‐TGCG‐48 bp‐GTGAGA GATGTCTGTCGATCCTCAA‐68 bp‐TTCAGTCTCCTCAACTCACCCATCCCGTCCCCCACCCTGGAT—‐47 bp—‐‐AGA	WT 7/10 −185 bp 1/10 −56 bp 2/10

**TABLE 2:** The embryo transfer and generation of cloned foetuses.

No.	Recipients	Donor cells	No. of transferred embryos	Pregnancy (%)	Days of pregnancy	No. of foetuses (alive)
1	P678	C12	537	+	33	2 (1)
2	P656		537	−	−	−
3	P798		537	−	−	−
4	P803		537	−	−	−
Total		−	2148	1 (25.0%)	−	2 (1)

**TABLE 3:** The embryo transfer and generation of cloned piglets.

No.	Recipients	Donor cells	No. of transferred embryos	Pregnancy (%)	Days of pregnancy	No. of offspring (alive)
1	P688	C12F01	400	+	115	5 (3)
2	P811		560	−	−	−
3	P812		400	−	−	−
4	P848		445	−	−	−
5	P685		445	−	−	−
5	P807		445	−	−	−
7	P840		445	+	114	6 (5)
8	P666		477	−	−	−
9	P724		500	−	−	−
10	P821		500	−	−	−
11	P876		500	−	−	−
12	P880		484	+	115	3 (3)
13	P879		484	+	115	5 (5)
14	P922		484	−	−	−
15	P943		484	+	115	2 (2)
16	P923		484	−	−	−
17	P939		484	+	115	7 (6)
Total		−		6 (35.3%)	−	28 (24)

**TABLE 4:** Birth weight and health status of of cloned piglets.

No.	ID	Body weight (g)	Status at birth	Survival time (day)	Recipients
1	33dDN8TGC12F01P01	950	Healthy	—	P688
2	33dDN8TGC12F01P02	850	Healthy	—	
3	33dDN8TGC12F01P03	850	Healthy	44	
4	33dDN8TGC12F01P04	400	Stillbirth	0	
5	33dDN8TGC12F01P05	550	Mummy	0	
6	33dDN8TGC12F01P06	900	Healthy	—	P840
7	33dDN8TGC12F01P07	650	Stillbirth	0	
8	33dDN8TGC12F01P08	800	Healthy	—	
9	33dDN8TGC12F01P09	300	Deformed	2	
10	33dDN8TGC12F01P10	850	Healthy	—	
11	33dDN8TGC12F01P11	650	Healthy	—	
12	33dDN8TGC12F01P12	340	Healthy	7	P939
13	33dDN8TGC12F01P13	560	Healthy	76	
14	33dDN8TGC12F01P14	580	Deformed, stillbirth	0	
15	33dDN8TGC12F01P15	530	Healthy	88	
16	33dDN8TGC12F01P16	520	Healthy	—	
17	33dDN8TGC12F01P17	720	Healthy	—	
18	33dDN8TGC12F01P18	530	Healthy	—	
19	33dDN8TGC12F01P19	550	Healthy	—	P879
20	33dDN8TGC12F01P20	650	Healthy	—	
21	33dDN8TGC12F01P21	820	Healthy	—	
22	33dDN8TGC12F01P22	760	Healthy	—	
23	33dDN8TGC12F01P23	500	Healthy	—	
24	33dDN8TGC12F01P24	530	Healthy	87	P880
25	33dDN8TGC12F01P25	430	Healthy	—	
26	33dDN8TGC12F01P26	370	Healthy	—	
27	33dDN8TGC12F01P27	640	Healthy	1	P943
28	33dDN8TGC12F01P28	690	Healthy	—	

**TABLE 5:** Genotyping of cloned pigs by Sanger sequencing.

Gene	No. of pigs	Sequence	Mutation
GGTA1	WT	ATGAATGTCAAAGGAAGAGTGGTTCTGTCAATGCTGCTTGTCTCAACTGTAATGGTTGTGTTTTGGGAATACATCAACAG	
	P1	ATGAATGT **‐‐‐‐‐‐‐‐‐‐‐‐‐‐‐‐‐‐‐‐‐‐‐‐‐‐‐‐‐‐‐‐‐‐‐‐‐‐‐‐‐‐‐‐‐‐‐‐‐‐‐‐** TTTTGGGAATACATCAACAG	−52 bp
	P2	ATGAATGT **‐‐‐‐‐‐‐‐‐‐‐‐‐‐‐‐‐‐‐‐‐‐‐‐‐‐‐‐‐‐‐‐‐‐‐‐‐‐‐‐‐‐‐‐‐‐‐‐‐‐‐‐** TTTTGGGAATACATCAACAG	−52 bp
	P3	ATGAATGT **‐‐‐‐‐‐‐‐‐‐‐‐‐‐‐‐‐‐‐‐‐‐‐‐‐‐‐‐‐‐‐‐‐‐‐‐‐‐‐‐‐‐‐‐‐‐‐‐‐‐‐‐** TTTTGGGAATACATCAACAG	−52 bp
	P5	ATGAATGT **—‐———‐‐‐‐‐‐‐‐‐—‐‐—‐‐‐‐‐‐‐‐‐‐‐‐‐‐‐‐‐‐‐‐‐‐** TTTTGGGAATACATCAACAG	−52 bp
	P8	ATGAATGT **‐‐‐‐‐‐‐‐‐‐‐‐‐‐‐‐‐‐‐‐‐‐‐‐‐‐‐‐‐‐‐‐‐‐‐‐‐‐‐‐‐‐‐‐‐‐‐‐‐‐‐‐** TTTTGGGAATACATCAACAG	−52 bp
	P9	ATGAATGT **‐‐‐‐‐‐‐‐‐‐‐‐‐‐‐‐‐‐‐‐‐‐‐‐‐‐‐‐‐‐‐‐‐‐‐‐‐‐‐‐‐‐‐‐‐‐‐‐‐‐‐‐** TTTTGGGAATACATCAACAG	−52 bp 20/20
	P12	ATGAATGT **‐‐‐‐‐‐‐‐‐‐‐‐‐‐‐‐‐‐‐‐‐‐‐‐‐‐‐‐‐‐‐‐‐‐‐‐‐‐‐‐‐‐‐‐‐‐‐‐‐‐‐‐** TTTTGGGAATACATCAACAG	−52 bp 10/10
	P24	ATGAATGT **‐‐‐‐‐‐‐‐‐‐‐‐‐‐‐‐‐‐‐‐‐‐‐‐‐‐‐‐‐‐‐‐‐‐‐‐‐‐‐‐‐‐‐‐‐‐‐‐‐‐‐‐** TTTTGGGAATACATCAACAG	−52 bp 10/10
CMAH	WT	AACAGATCACGTACCTTACTCAC(60 bp)CCTGCTTTTGCGCGAGGATGGTGGTTACTACACGAGCCTCCATCTGATTGGCTG	
	P1	AAC **‐** GATCACGTACCTTACTCAC(60 bp)CCTGCTTTTGCGCGAGGATGGTGGTTACTACACGAGCCTCCA **‐‐‐** GATTGGCTG AACAGAT **‐‐‐‐‐‐‐‐‐‐‐‐‐‐‐‐** (60 bp) **‐‐‐‐‐‐‐‐‐‐‐‐‐‐‐‐‐‐‐‐‐‐‐‐‐‐‐‐‐‐‐‐‐‐‐‐‐‐‐‐‐‐‐‐‐‐‐‐** TGGCTG	−4 bp 4/5 −124 bp 1/5
	P2	AAC **‐** GATCACGTACCTTACTCAC(60 bp)CCTGCTTTTGCGCGAGGATGGTGGTTACTACACGAGCCTCCA **‐‐‐** GATTGGCTG AACAGAT **‐‐‐‐‐‐‐‐‐‐‐‐‐‐‐‐** (60 bp) **‐‐‐‐‐‐‐‐‐‐‐‐‐‐‐‐‐‐‐‐‐‐‐‐‐‐‐‐‐‐‐‐‐‐‐‐‐‐‐‐‐‐‐‐‐‐‐‐** TGGCTG	−4 bp 5/6 −124 bp 1/6
	P3	AAC **‐** GATCACGTACCTTACTCAC(60 bp)CCTGCTTTTGCGCGAGGATGGTGGTTACTACACGAGCCTCCA **‐‐‐** GATTGGCTG AACAGAT **‐‐‐‐‐‐‐‐‐‐‐‐‐‐‐‐** (60 bp) **‐‐‐‐‐‐‐‐‐‐‐‐‐‐‐‐‐‐‐‐‐‐‐‐‐‐‐‐‐‐‐‐‐‐‐‐‐‐‐‐‐‐‐‐‐‐‐‐** TGGCTG	−4 bp 8/11 −124 bp 3/11
	P5	AAC **‐** GATCACGTACCTTACTCAC(60 bp)CCTGCTTTTGCGCGAGGATGGTGGTTACTACACGAGCCTCCA **‐‐‐** GATTGGCTG	−4 bp 6/6
	P8	AAC **‐** GATCACGTACCTTACTCAC(60 bp)CCTGCTTTTGCGCGAGGATGGTGGTTACTACACGAGCCTCCA **‐‐‐** GATTGGCTG AACAGAT **‐‐‐‐‐‐‐‐‐‐‐‐‐‐‐‐** (60 bp) **‐‐‐‐‐‐‐‐‐‐‐‐‐‐‐‐‐‐‐‐‐‐‐‐‐‐‐‐‐‐‐‐‐‐‐‐‐‐‐‐‐‐‐‐‐‐‐‐** TGGCTG	−4 bp 3/6 −124 bp 3/6
	P9	AAC **‐** GATCACGTACCTTACTCAC(60 bp)CCTGCTTTTGCGCGAGGATGGTGGTTACTACACGAGCCTCCA **‐‐‐** GATTGGCTG AACAGAT **‐‐‐‐‐‐‐‐‐‐‐‐‐‐‐‐** (60 bp) **‐‐‐‐‐‐‐‐‐‐‐‐‐‐‐‐‐‐‐‐‐‐‐‐‐‐‐‐‐‐‐‐‐‐‐‐‐‐‐‐‐‐‐‐‐‐‐‐** TGGCTG	−4 bp 7/10 −124 bp 3/10
	P12	AAC **‐** GATCACGTACCTTACTCAC(60 bp)CCTGCTTTTGCGCGAGGATGGTGGTTACTACACGAGCCTCCA **‐‐‐** GATTGGCTG AACAGAT **‐‐‐‐‐‐‐‐‐‐‐‐‐‐‐‐** (60 bp) **‐‐‐‐‐‐‐‐‐‐‐‐‐‐‐‐‐‐‐‐‐‐‐‐‐‐‐‐‐‐‐‐‐‐‐‐‐‐‐‐‐‐‐‐‐‐‐‐** TGGCTG	−4 bp 7/10 −124 bp 3/10
	P24	AAC **‐** GATCACGTACCTTACTCAC(60 bp)CCTGCTTTTGCGCGAGGATGGTGGTTACTACACGAGCCTCCA **‐‐‐** GATTGGCTG AACAGAT **‐‐‐‐‐‐‐‐‐‐‐‐‐‐‐‐** (60 bp) **‐‐‐‐‐‐‐‐‐‐‐‐‐‐‐‐‐‐‐‐‐‐‐‐‐‐‐‐‐‐‐‐‐‐‐‐‐‐‐‐‐‐‐‐‐‐‐‐** TGGCTG	−4 bp 4/8 −124 bp 4/8
β4GalNT2	WT	GATGTCTGTCGATCCTCA(110 bp)GATGCGCAGACGCTGAAGCTTCTACCTGAGAA(21 bp)CTACAGTGGAATCTGGTGGTGAGA GATGTCTGTCGATCCTCA(110 bp)GATGCGCAGACGCTGAAGCTTCTACCTGAGAA(9 bp)CTACGGTGAAAACGGGTGAGA	
	P1	GATG **‐‐‐‐‐‐‐‐‐‐‐‐‐‐** (110 bp) **‐** ATGCGCAGACGCTGAAGCTTCTACCTGAGAA(21 bp)CTACAGTGGAATCTGGTGGTGAGA GATGTCTGTCGATCCTCA(110 bp)GA **‐‐‐‐‐‐‐‐‐‐‐‐‐‐‐‐‐‐‐‐‐‐‐‐‐‐‐‐‐‐‐** (9 bp)‐ **‐‐‐‐‐‐‐‐‐‐‐‐‐‐‐‐‐‐** GA	−125 bp 3/5 −57 bp 2/5
	P2	GAT **‐‐‐‐‐‐‐‐‐‐‐‐‐‐‐** (110 bp) **‐‐** TGCGCAGACGCTGAAGCTTCTACCTGAGAA(21 bp)CTACAGTGGAATCTGGTGGTGAGA GATG **‐‐‐‐‐‐‐‐‐‐‐‐‐‐** (110 bp) **‐** ATGCGCAGACGCTGAAGCTTCTACCTGAGAA(21 bp)CTACAGTGGAATCTGGTGGTGAGA GAT **‐‐‐‐‐‐‐‐‐‐‐‐‐‐** A(110 bp)GAT **‐‐‐‐‐‐‐‐‐‐‐‐‐‐‐‐‐‐‐‐‐‐‐‐‐‐‐‐‐‐** (9 bp)‐ **‐‐‐‐‐‐‐‐‐‐‐‐‐‐‐‐‐‐** GA GATGTCTGTCGATCCTCA(110 bp)GA **‐‐‐‐‐‐‐‐‐‐‐‐‐‐‐‐‐‐‐‐‐‐‐‐‐‐‐‐‐‐‐** (9 bp)‐ **‐‐‐‐‐‐‐‐‐‐‐‐‐‐‐‐‐‐** GA	−127 bp 2/6 −125 bp 2/6 −70 bp 1/6 −57 bp 1/6
	P3	GAT **‐‐‐‐‐‐‐‐‐‐‐‐‐‐‐** (110 bp) **‐‐** TGCGCAGACGCTGAAGCTTCTACCTGAGAA(21 bp)CTACAGTGGAATCTGGTGGTGAGA GATG **‐‐‐‐‐‐‐‐‐‐‐‐‐‐** (110 bp) **‐** ATGCGCAGACGCTGAAGCTTCTACCTGAGAA(21 bp)CTACAGTGGAATCTGGTGGTGAGA GAT **‐‐‐‐‐‐‐‐‐‐‐‐‐‐** A(110 bp)GAT **—‐‐‐‐‐‐‐‐‐‐‐‐‐‐‐‐‐‐‐‐‐‐‐‐‐‐‐** (9 bp)‐ **—‐‐‐‐‐‐‐‐‐‐‐‐‐‐‐** GA	−127 bp 5/8 −125 bp 1/8 −70 bp 2/8
	P5	GAT **‐‐‐‐‐‐‐‐‐‐‐‐‐‐‐** (110 bp) **‐‐** TGCGCAGACGCTGAAGCTTCTACCTGAGAA(21 bp)CTACAGTGGAATCTGGTGGTGAGA GATG **‐‐‐‐‐‐‐‐‐‐‐‐‐‐** (110 bp) **‐** ATGCGCAGACGCTGAAGCTTCTACCTGAGAA(21 bp)CTACAGTGGAATCTGGTGGTGAGA GATGTCTGTCGATCCTCA(110 bp)GA **‐‐‐‐‐‐‐‐‐‐‐‐‐‐‐‐‐‐‐‐‐‐‐‐‐‐‐‐‐‐‐** (9 bp)‐ **‐‐‐‐‐‐‐‐‐‐‐‐‐‐‐‐‐‐** GA	−127 bp 3/8 −125 bp 3/8 −57 bp 2/8
	P8	GAT **‐‐‐‐‐‐‐‐‐‐‐‐‐‐‐** (110 bp) **‐‐** TGCGCAGACGCTGAAGCTTCTACCTGAGAA(21 bp)CTACAGTGGAATCTGGTGGTGAGA GATG **‐‐‐‐‐‐‐‐‐‐‐‐‐‐** (110 bp) **‐** ATGCGCAGACGCTGAAGCTTCTACCTGAGAA(21 bp)CTACAGTGGAATCTGGTGGTGAGA GAT **‐‐‐‐‐‐‐‐‐‐‐‐‐‐** A(110 bp)GAT **‐‐‐‐‐‐‐‐‐‐‐‐‐‐‐‐‐‐‐‐‐‐‐‐‐‐‐‐‐‐** (9 bp)‐ **‐‐‐‐‐‐‐‐‐‐‐‐‐‐‐‐‐‐** GA GATGTCTGTCGATCCTCA(110 bp)GA **‐‐‐‐‐‐‐‐‐‐‐‐‐‐‐‐‐‐‐‐‐‐‐‐‐‐‐‐‐‐‐** (9 bp)‐ **‐‐‐‐‐‐‐‐‐‐‐‐‐‐‐‐‐‐** GA	−127 bp 1/7 −125 bp 2/7 −70 bp 2/8 −57 bp 3/7
	P9	GAT **‐‐‐‐‐‐‐‐‐‐‐‐‐‐‐** (110 bp) **‐‐** TGCGCAGACGCTGAAGCTTCTACCTGAGAA(21 bp)CTACAGTGGAATCTGGTGGTGAGA GATG **‐‐‐‐‐‐‐‐‐‐‐‐‐‐** (110 bp) **‐** ATGCGCAGACGCTGAAGCTTCTACCTGAGAA(21 bp)CTACAGTGGAATCTGGTGGTGAGA GAT **‐‐‐‐‐‐‐‐‐‐‐‐‐‐** A(110 bp)GAT **‐‐‐‐‐‐‐‐‐‐‐‐‐‐‐‐‐‐‐‐‐‐‐‐‐‐‐‐‐‐** (9 bp)‐ **‐‐‐‐‐‐‐‐‐‐‐‐‐‐‐‐‐‐** GA	−127 bp 5/18 −125 bp 8/18 −70 bp 5/18
	P12	GAT **‐‐‐‐‐‐‐‐‐‐‐‐‐‐‐** (110 bp) **‐‐** TGCGCAGACGCTGAAGCTTCTACCTGAGAA(21 bp)CTACAGTGGAATCTGGTGGTGAGA GATG **‐‐‐‐‐‐‐‐‐‐‐‐‐‐** (110 bp) **‐** ATGCGCAGACGCTGAAGCTTCTACCTGAGAA(21 bp)CTACAGTGGAATCTGGTGGTGAGA GAT **‐‐‐‐‐‐‐‐‐‐‐‐‐‐** A(110 bp)GAT **‐‐‐‐‐‐‐‐‐‐‐‐‐‐‐‐‐‐‐—‐‐‐‐‐‐‐‐** (9 bp)‐ **‐‐‐‐‐‐‐‐‐‐‐‐‐‐‐‐‐‐** GA GATGTCTGTCGATCCTCA(110 bp)GA **—‐‐‐‐‐‐‐‐‐‐‐‐‐‐‐‐‐‐‐‐‐‐‐‐‐‐‐‐** (9 bp)‐ **‐‐‐‐‐‐‐‐‐‐‐‐‐‐‐‐‐‐** GA	−127 bp 8/18 −125 bp 4/18 −70 bp 5/18 −57 bp 1/18
	P24	GAT **‐‐‐‐‐‐‐‐‐‐‐‐‐‐‐** (110 bp) **‐‐** TGCGCAGACGCTGAAGCTTCTACCTGAGAA(21 bp)CTACAGTGGAATCTGGTGGTGAGA GATG **‐‐‐‐‐‐‐‐‐‐‐‐‐‐** (110 bp) **‐** ATGCGCAGACGCTGAAGCTTCTACCTGAGAA(21 bp)CTACAGTGGAATCTGGTGGTGAGA GAT **—‐‐‐‐‐‐‐‐‐‐‐** A(110 bp)GAT **—‐‐‐‐‐‐‐‐‐‐‐‐‐‐‐‐‐‐‐‐‐‐‐‐‐‐‐** (9 bp)‐ **‐‐‐‐‐‐‐‐‐‐‐‐‐‐‐‐‐‐** GA GATGTCTGTCGATCCTCA(110 bp)GAT **‐‐‐‐‐‐‐‐‐‐‐‐‐‐‐‐‐‐‐‐‐‐‐‐‐‐‐‐‐‐** (9 bp)‐ **‐‐‐‐‐‐‐‐‐‐‐‐‐‐‐‐‐‐** GA	−127 bp 5/15 −125 bp 6/15 −70 bp 3/15 −56 bp 1/15

### 3.2 Phenotypic Identification of 8‐GEC Pigs and F1 Offspring

**FIGURE 4:**
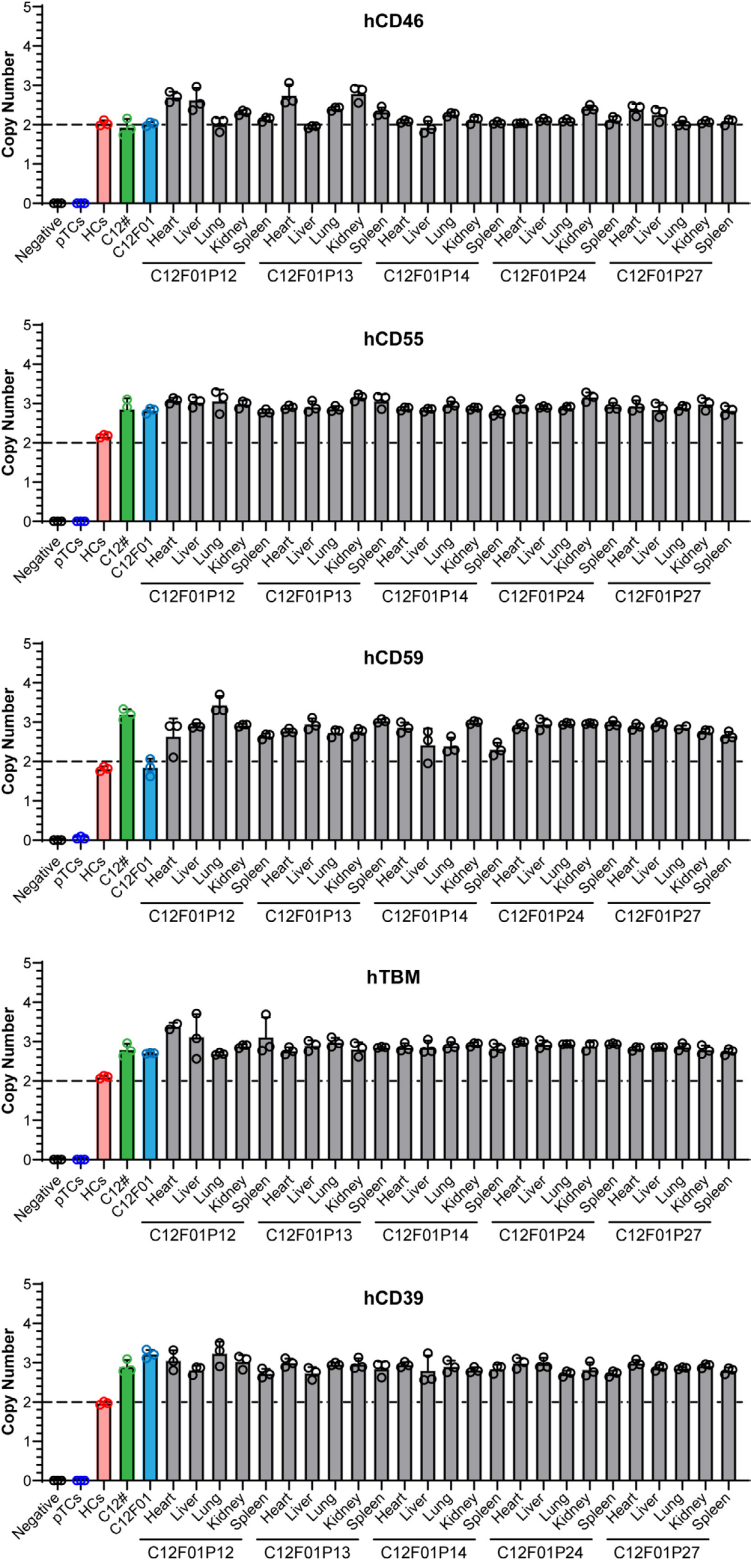
Copy numbers of transgenes hCD46, hCD55, hCD59, hTBM and hCD39 in donor cell lines, cloned fetus, and piglets. The copy numbers of all transgenes were detected by ddPCR. Negative: Water, and pTCs. HCs = human cells; pTCs = pre‐transfected cells.

**FIGURE 5:**
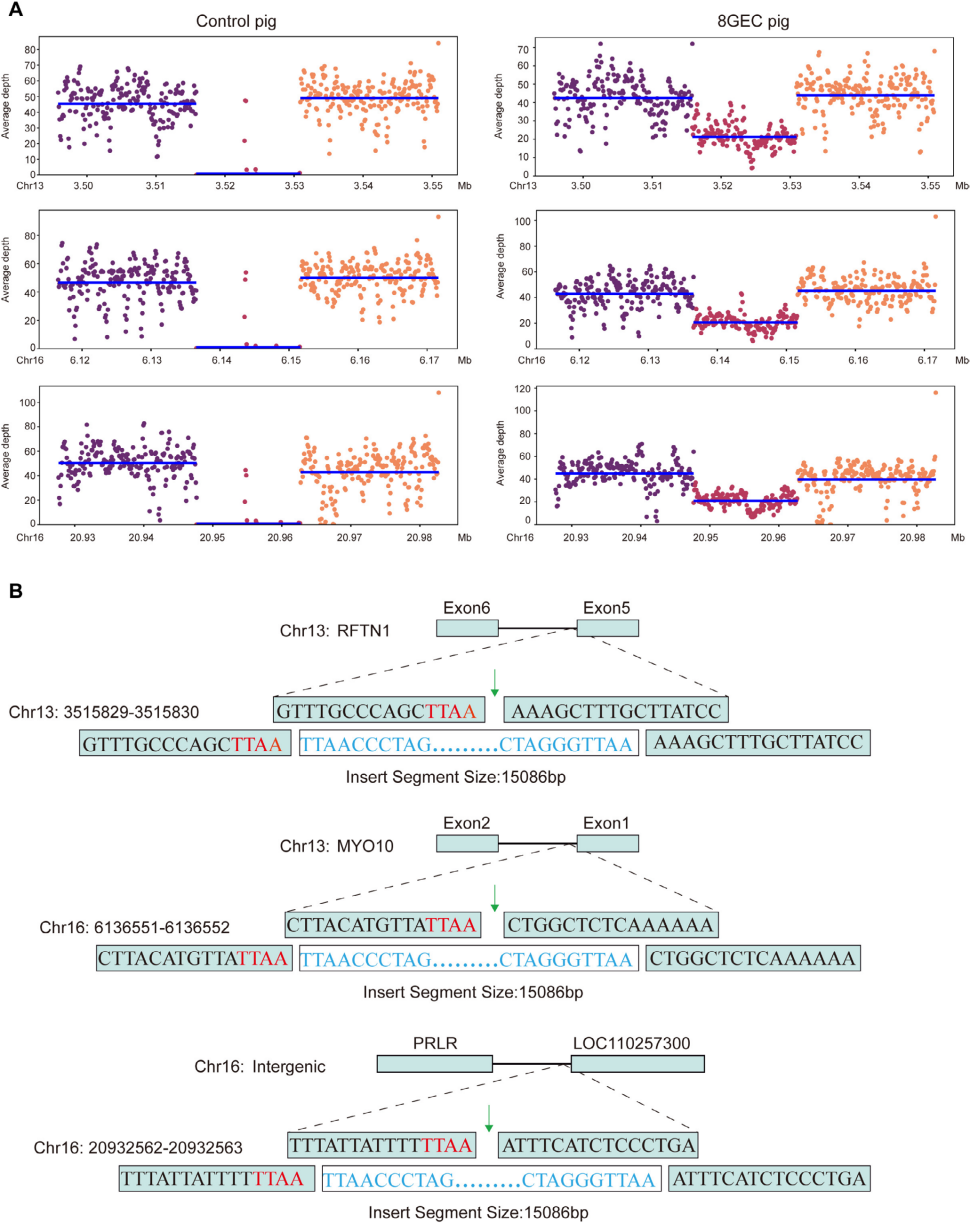
Analysis of transgenic insertion by WGS A. Confirmation of transgenic copies and insertion location. It was confirmed that the 8‐GEC pig transgenic copies were 3 and all were complete through whole genome sequencing analysis. The average depth of insertion fragments in WT pigs is 0, while the average depth of insertion fragments in 8‐GEC pigs is about 20 and the average depth of the pig's own genome is about 40, which means that the average depth of insertion fragments is half of the depth of the pig's own genome. Namely, the pig genome is default diploid, so the insertion fragment is haploid. The final confirmation of the insertion fragment is 3 copies. Moreover, in all three copies, the average depth is around 20, indicating that the entire insertion fragment is intact. The horizontal axis represents the position of the chromosome, which is divided into three segments. The left part is the pig genome sequence (20 kb) at upstream of insertion fragment; The middle part is the insertion fragment; and the right part is the pig genome sequence (20 kb) at downstream of the inserted fragment. The vertical axis represents the average depth. Each point represents the average depth of a window of 100 bp, and the blue line represents the average depth of that fragment. A total of (*n* = 6) 8‐GEC pigs were used for WGS and here in (A) is the data of one head, while the data of remaining 5 heads is shown in Figure S2. (B) Schematic diagram of the transgenic insertion position. Three copies of transgenes are inserted into the introns of pig RFTN1 and MYO10 genes, as well as the intergenic region between PRLR and LOC110257300 genes, respectively.

**FIGURE 6:**
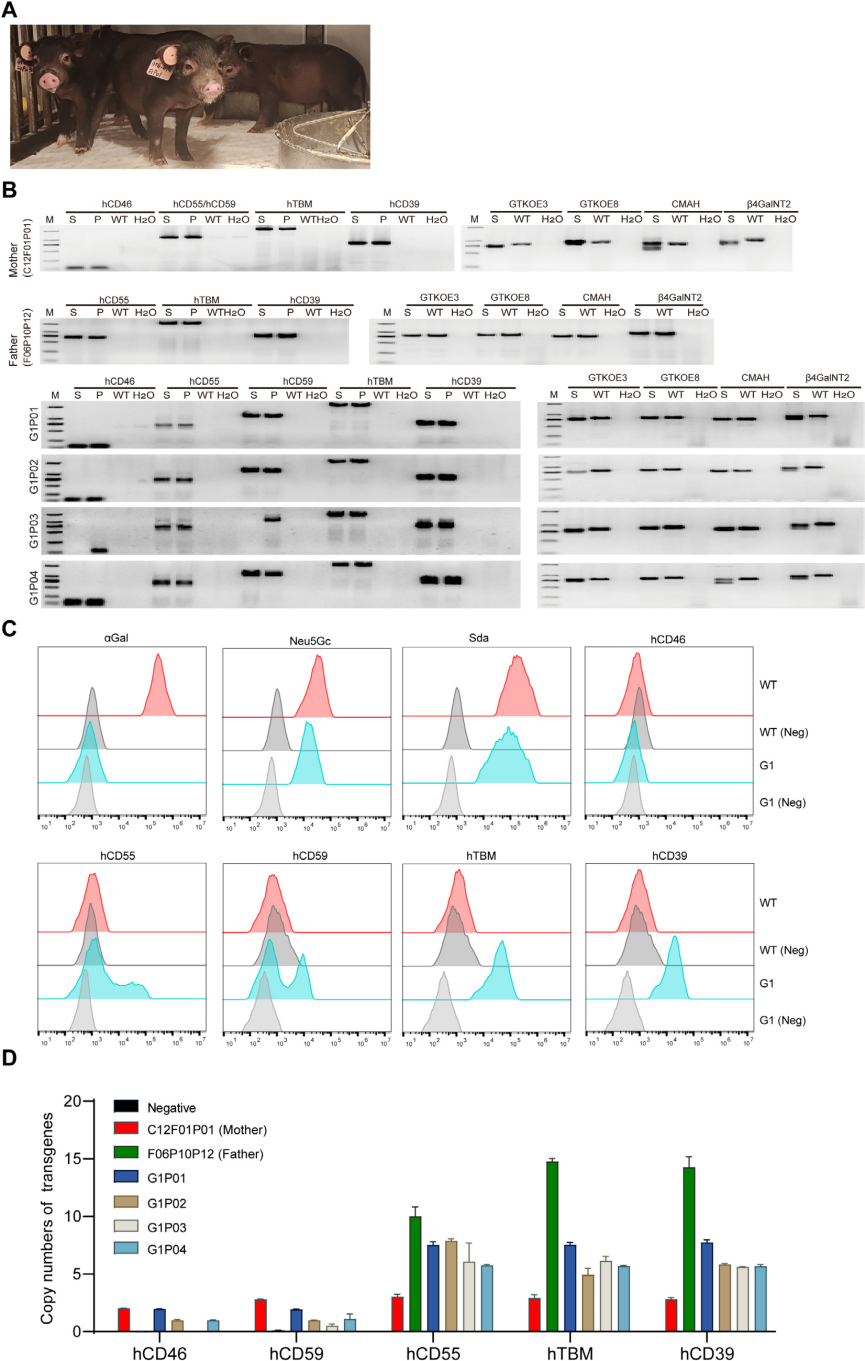
Identification of F1 generation offspring of 8‐GEC pig. (A) Photo of F1 generation piglets. (B) Identification of 8 genes by PCR. (C) The expression of αGal, Neu5Gc, Sda, hCD46, hCD55, hCD59, hTBM and hCD39 in umbilical vein endothelial cells was confirmed by flow cytometry. (D) Copy numbers of transgenes hCD46, hCD55, hCD59, hTBM, and hCD39 in F1 generation piglets. The offspring were generated by natural mating of 8 GE female pigs with previously generated 4 GE male pigs (GTKO/hCD55/hTBM/hCD39) [21].

To identify the expression and function of 8 modified genes, we performed qPCR, flow cytometry, immunofluorescence, antibody binding assay and complement‐dependent cytotoxicity assay. The results of mRNA expression levels showed that all transgenes were expressed in the heart, kidney, liver and lung tissues of cloned piglets. The mRNA expression levels of hCD46 and hCD39 were higher, while those of hCD55, hCD59 and hTBM were lower in the kidney of 8‐GEC pigs. It is worth noting that all genes exhibited higher expression in lung tissues, while lower expression in heart tissue (Figure 3B–F). The result of flow cytometry showed that αGal, Neu5Gc and Sda were deficient, while hCD46, hCD55, hCD59, hTBM and hCD39 were expressed in the aortic endothelial cells of cloned piglets (Figure 3G). The result of immunofluorescence also revealed that the protein expression of these 5 transgenes was expressed in cloned piglet's kidney (Figure 3H). In addition, we found that the protein expression levels of the same gene in different tissues of the same cloned individual (P03) were not consistent (Figure S1). Nevertheless, when rhesus monkey serum was used to perform antigen–antibody binding, and complement‐dependent cytotoxicity assays, the binding ability of monkey IgG and IgM to 8‐GEC pigs' PBMCs was significantly reduced (Figure 3I,J), and complement attack of porcine PBMCs was significantly weakened (Figure 3K). The PiggyBac transposon system integrates exogenous genes into the genome at ‘TTAA’ box in a random manner, which can result in multiple transgenic copies being integrated into the genome simultaneously, potentially disrupting functional genes [29]. Therefore, we determined the copy number of 5 transgenes in heart, liver, lung, kidney and spleen tissues of 8‐GEC pigs by ddPCR. Among them, the copy number of hCD55, hCD59, hTBM and hCD39 genes was 3, while the copy number of the hCD46 gene was 2 in most samples (Figure 4), which might be caused by incomplete insertion of sequence. To verify this, we performed an analysis of transgenic insertion position through WGS. The results showed that the transgenic sequence was complete, and all of the transgenes had 3 copies (Figure 5A, Figure S2), which are inserted into the introns of pig RFTN1 and MYO10 genes, as well as the intergenic region between PRLR and LOC110257300 genes (Figure 5B, Table S5). Therefore, we considered that the copy numbers detected by ddPCR were limited by the sensitivity of probe and primer. In summary, we successfully generated the 8 (GTKO/CMAHKO/β4GalNT2KO/hCD46/hCD55/hCD59/hTBM/hCD39) GEC pigs by gene editing and somatic cell cloning technology. So far, one 8‐GEC female pig was mated with an already generated 4‐GE (GTKO/hCD55/hTBM/hCD39) male pig [21] that delivered four viable F1 generation individuals (Figure 6A, Table 6). In F1 offspring, all of them were biallelic KO on GGTA1 and monoallelic KO on CMAH and β4GalNT2. Three of them carried hCD46, hCD55, hCD59 hTBM, and hCD39. The remaining one (G1P03) carried hCD55, hTBM and hCD39 (Figure 6B, Tables 7 and 8). The flow cytometry showed that αGal was deficient, but Neu5Gc and Sda were expressed. The expression of hCD55, hCD59, hTBM, and hCD39 was detectable in the umbilical vein endothelial cells of F1 piglet, but the expression of hCD46 was not observed (Figure 6C). The copy numbers of hCD46 and hCD59 genes in the four offspring were less than three of the mothers, while the copy numbers of the hCD55, hTBM and hCD39 genes were between those of the mother and father (Figure 6D). Collectively, these GEC pigs have the ability for germline transmission.

**TABLE 6:** Production of F1 generation offspring of 8‐GEC pigs.

No.	F1 generation	Body weight (g)	Status at birth	Gender
1	G1P01	460	Healthy	♂
2	G1P02	460	Healthy	♂
3	G1P03	550	Healthy	♂
4	G1P04	570	Healthy	♂

*Note*: The offspring were generated by natural mating of 8 GE female pigs with previously generated 4 GE male pigs (GTKO/hCD55/hTBM/hCD39) [21].

Abbreviation: GE = gene edited.

**TABLE 7:** Genotyping of F1 generation offspring of 8‐GEC pigs.

Gene	No. of pigs	Sequence	Mutation
GGTA1 on Exon 3	WT	TGATGTATTCCCAAAACACAACCATTACAGTTGAGACAAGCAGCATTGACAGAACCACTCTTCCTTTGACATTCATTATTTTCTCCTGGGAAAAGAAAAG	WT
	4 GE	TGATGTATTCCCAAAACACAACCATTACAGTTGAGACAAGCAGCATTGACAGAACCACTCTTCCTTTGACATTCATTATTTTCTCCTGGGAAAAGAAAAG	WT
	8 GE	TGATGTATTCCCAAAA‐‐‐‐‐‐‐‐‐‐‐‐‐‐‐‐‐‐‐‐‐‐‐‐‐‐‐‐‐‐‐‐‐‐‐‐‐‐‐‐‐‐‐‐‐‐‐‐‐‐‐‐‐ACATTCATTATTTTCTCCTGGGAAAAGAAAAG	−52 bp
	G1P01	TGATGTATTCCCAAAACACAACCATTACAGTTGAGACAAGCAGCATTGACAGAACCACTCTTCCTTTGACATTCATTATTTTCTCCTGGGAAAAGAAAAG	WT 2/4
		TGATGTATTCCCAAAA‐‐‐‐‐‐‐‐‐‐‐‐‐‐‐‐‐‐‐‐‐‐‐‐‐‐‐‐‐‐‐‐‐‐‐‐‐‐‐‐‐‐‐‐‐‐‐‐‐‐‐‐‐ACATTCATTATTTTCTCCTGGGAAAAGAAAAG	−52 bp 2/4
	G1P02	TGATGTATTCCCAAAACACAACCATTACAGTTGAGACAAGCAGCATTGACAGAACCACTCTTCCTTTGACATTCATTATTTTCTCCTGGGAAAAGAAAAG	WT 4/8
		TGATGTATTCCCAAAA‐‐‐‐‐‐‐‐‐‐‐‐‐‐‐‐‐‐‐‐‐‐‐‐‐‐‐‐‐‐‐‐‐‐‐‐‐‐‐‐‐‐‐‐‐‐‐‐‐‐‐‐‐ACATTCATTATTTTCTCCTGGGAAAAGAAAAG	−52 bp 4/8
	G1P03	TGATGTATTCCCAAAACACAACCATTACAGTTGAGACAAGCAGCATTGACAGAACCACTCTTCCTTTGACATTCATTATTTTCTCCTGGGAAAAGAAAAG	WT 4/11
		TGATGTATTCCCAAAA‐‐‐‐‐‐‐‐‐‐‐‐‐‐‐‐‐‐‐‐‐‐‐‐‐‐‐‐‐‐‐‐‐‐‐‐‐‐‐‐‐‐‐‐‐‐‐‐‐‐‐‐‐ACATTCATTATTTTCTCCTGGGAAAAGAAAAG	−52 bp 7/11
	G1P04	TGATGTATTCCCAAAACACAACCATTACAGTTGAGACAAGCAGCATTGACAGAACCACTCTTCCTTTGACATTCATTATTTTCTCCTGGGAAAAGAAAAG	WT 3/9
		TGATGTATTCCCAAAA‐‐‐‐‐‐‐‐‐‐‐‐‐‐‐‐‐‐‐‐‐‐‐‐‐‐‐‐‐‐‐‐‐‐‐‐‐‐‐‐‐‐‐‐‐‐‐‐‐‐‐‐‐ACATTCATTATTTTCTCCTGGGAAAAGAAAAG	−52 bp 6/9
GGTA1 on Exon 8	WT	ATGATGCGCATGAAGACCATCGGGGAGCACATCCTGGC(90)GGTGGCTCAGCTACAGGCCTGGTGGTACAAGGCACATCCTGACGAG	WT
	4 GE	ATGCG‐‐‐‐‐‐‐‐‐‐‐‐‐‐‐‐‐‐‐‐‐‐‐‐‐‐CATCCTGGC(90)GGTGGCTCAGCTTACAGGCCTGGTGGTACAAGGCACATCCTGACGAG	+3 bp/−26 bp
		ATGATGCGCATGAAGA‐‐‐TCGGGGAGCACATCCTGGC(90)GGTGGCTCAGCTTACAGGCCTGGTGGTACAAGGCACATCCTGACGAG	+1 bp/−3 bp
	8 GE	ATGATGCGCATGAAGACCATCGGGGAGCACATCCTGGC(90)GGTGGCTCAGCTACAGGCCTGGTGGTACAAGGCACATCCTGACGAG	WT
	G1P01	ATGATGCGCATGAAGACCATCGGGGAGCACATCCTGGC(90)GGTGGCTCAGCTACAGGCCTGGTGGTACAAGGCACATCCTGACGAG	WT 1/6
		ATGCG‐‐‐‐‐‐‐‐‐‐‐‐‐‐‐‐‐‐‐‐‐‐‐‐‐‐CATCCTGGC(90)GGTGGCTCAGCTTACAGGCCTGGTGGTACAAGGCACATCCTGACGAG	+3 bp/−26 bp 5/6
	G1P02	ATGATGCGCATGAAGACCATCGGGGAGCACATCCTGGC(90)GGTGGCTCAGCTACAGGCCTGGTGGTACAAGGCACATCCTGACGAG	WT 2/5
		ATGCG‐‐‐‐‐‐‐‐‐‐‐‐‐‐‐‐‐‐‐‐‐‐‐‐‐‐CATCCTGGC(90)GGTGGCTCAGCTTACAGGCCTGGTGGTACAAGGCACATCCTGACGAG	+3 bp/−26 bp 3/5
	G1P03	ATGATGCGCATGAAGACCATCGGGGAGCACATCCTGGC(90)GGTGGCTCAGCTACAGGCCTGGTGGTACAAGGCACATCCTGACGAG	WT 6/10
		ATGCG‐‐‐‐‐‐‐‐‐‐‐‐‐‐‐‐‐‐‐‐‐‐‐‐‐‐CATCCTGGC(90)GGTGGCTCAGCTTACAGGCCTGGTGGTACAAGGCACATCCTGACGAG	+3 bp/−26 bp 4/10
	G1P04	ATGATGCGCATGAAGACCATCGGGGAGCACATCCTGGC(90)GGTGGCTCAGCTACAGGCCTGGTGGTACAAGGCACATCCTGACGAG	WT 6/9
		ATGCG‐‐‐‐‐‐‐‐‐‐‐‐‐‐‐‐‐‐‐‐‐‐‐‐‐‐CATCCTGGC(90)GGTGGCTCAGCTTACAGGCCTGGTGGTACAAGGCACATCCTGACGAG	+3 bp/−26 bp 3/9
CMAH	WT	TCACTGTCTTCCAACAGATCACGTACCTTACTCACG(84)TACTACACGAGCCTCCATCTGATTGG(62)GTAAGGAAGGGTGAGCCCTCAACTCCGAAGA	WT
	4 GE	TCACTGTCTTCCAACAGATCACGTACCTTACTCACG(84)TACTACACGAGCCTCCATCTGATTGG(62)GTAAGGAAGGGTGAGCCCTCAACTCCGAAGA	WT
	8 GE	TCACTGTCTTCCAAC‐GATCACGTACCTTACTCACG(84)TACTACACGAGCCTCCA‐‐‐GATTGG(62)GTAAGGAAGGGTGAGCCCTCAACTCCGAAGA	−4 bp
		TCACTGTCTTCCAACAGAT‐‐‐‐‐‐‐‐‐‐‐‐‐‐‐‐‐**(84)**‐‐‐‐‐‐‐‐‐‐‐‐‐‐‐‐‐‐‐‐‐‐‐TGG(62)GTAAGGAAGGGTGAGCCCTCAACTCCGAAGA	−124 bp
	G1P01	TCACTGTCTTCCAACAGATCACGTACCTTACTCACG(84)TACTACACGAGCCTCCATCTGATTGG(62)GTAAGGAAGGGTGAGCCCTCAACTCCGAAGA	WT 5/7
		TCACTGTCTTCCAAC‐GATCACGTACCTTACTCACG(84)TACTACACGAGCCTCCA‐‐‐GATTGG(62)GTAAGGAAGGGTGAGCCCTCAACTCCGAAGA	−4 bp 2/7
	G1P02	TCACTGTCTTCCAACAGATCACGTACCTTACTCACG(84)TACTACACGAGCCTCCATCTGATTGG(62)GTAAGGAAGGGTGAGCCCTCAACTCCGAAGA	WT 3/8
		TCACTGTCTTCCAAC‐GATCACGTACCTTACTCACG(84)TACTACACGAGCCTCCA‐‐‐GATTGG(62)GTAAGGAAGGGTGAGCCCTCAACTCCGAAGA	−4 bp 5/8
	G1P03	TCACTGTCTTCCAACAGATCACGTACCTTACTCACG(84)TACTACACGAGCCTCCATCTGATTGG(62)GTAAGGAAGGGTGAGCCCTCAACTCCGAAGA	WT 2/8
		TCACTGTCTTCCAAC‐GATCACGTACCTTACTCACG(84)TACTACACGAGCCTCCA‐‐‐GATTGG(62)GTAAGGAAGGGTGAGCCCTCAACTCCGAAGA	−4 bp 6/8
	G1P04	TCACTGTCTTCCAACAGATCACGTACCTTACTCACG(84)TACTACACGAGCCTCCATCTGATTGG(62)GTAAGGAAGGGTGAGCCCTCAACTCCGAAGA	WT 1/7
		TCACTGTCTTCCAACAGAT‐‐‐‐‐‐‐‐‐‐‐‐‐‐‐‐‐**(84)**‐‐‐‐‐‐‐‐‐‐‐‐‐‐‐‐‐‐‐‐‐‐‐TGG(62)GTAAGGAAGGGTGAGCCCTCAACTCCGAAGA	−124 bp 6/7
β4GalNT2	WT	AGCCCTAGATGTCTGTCGATCCTCAAGAT(99)CACCCTGGATGCGCAGACGCTGAAGCTTCTACC(15)CTACGGTGAAAACGGGTGAGATGGCAA	WT
		AGCCCTAGATGTCTGTCGATCCTCAAGAT(99)CACCCTGGATGCGCAGACGCTGAAGCTTCTACC(27)CTACAGTGGAATCTGGTGAGATGGCAA	WT
	4 GE	AGCCCTAGATGTCTGTCGATCCTCAAGAT(99)CACCCTGGATGCGCAGACGCTGAAGCTTCTACC(15)CTACGGTGAAAACGGGTGAGATGGCAA	WT
		AGCCCTAGATGTCTGTCGATCCTCAAGAT(99)CACCCTGGATGCGCAGACGCTGAAGCTTCTACC(27)CTACAGTGGAATCTGGTGAGATGGCAA	WT
	8 GE	AGCCCTAGAT‐‐‐‐‐‐‐‐‐‐‐‐‐‐‐‐‐‐‐**(99)**‐‐‐‐‐‐‐‐‐TGCGCAGACGCTGAAGCTTCTACC(27)CTACAGTGGAATCTGGTGAGATGGCAA	−127 bp
		AGCCCTAGAG‐‐‐‐‐‐‐‐‐‐‐‐‐‐‐‐‐‐**(99)**‐‐‐‐‐‐‐‐ATGCGCAGACGCTGAAGCTTCTACC(27)CTACAGTGGAATCTGGTGAGATGGCAA	−125 bp
		AGCCCTAGAT‐‐‐‐‐‐‐‐‐‐‐‐‐‐AAGAT(99)CACCCTGGAT‐‐‐‐‐‐‐‐‐‐‐‐‐‐‐‐‐‐‐‐‐‐‐**(15)**‐‐‐‐‐‐‐‐‐‐‐‐‐‐‐‐‐‐AGATGGCAA	−14 bp/−56 bp
		AGCCCTAGATGTCTGTCGATCCTCAAGAT(99)CACCCTGGA‐‐‐‐‐‐‐‐‐‐‐‐‐‐‐‐‐‐‐‐‐‐‐‐**(15)**‐‐‐‐‐‐‐‐‐‐‐‐‐‐‐‐‐‐AGATGGCAA	−57 bp
	G1P01	AGCCCTAGATGTCTGTCGATCCTCAAGAT(99)CACCCTGGATGCGCAGACGCTGAAGCTTCTACC(15)CTACGGTGAAAACGGGTGAGATGGCAA	WT 2/10
		AGCCCTAGAG‐‐‐‐‐‐‐‐‐‐‐‐‐‐‐‐‐‐**(99)**‐‐‐‐‐‐‐‐ATGCGCAGACGCTGAAGCTTCTACC(27)CTACAGTGGAATCTGGTGAGATGGCAA	−125 bp 3/10
		AGCCCTAGAT‐‐‐‐‐‐‐‐‐‐‐‐‐‐AAGAT(99)CACCCTGGAT‐‐‐‐‐‐‐‐‐‐‐‐‐‐‐‐‐‐‐‐‐‐‐**(15)**‐‐‐‐‐‐‐‐‐‐‐‐‐‐‐‐‐‐AGATGGCAA	−14 bp/−56 bp 5/10
	G1P02	AGCCCTAGATGTCTGTCGATCCTCAAGAT(99)CACCCTGGATGCGCAGACGCTGAAGCTTCTACC(15)CTACGGTGAAAACGGGTGAGATGGCAA	WT 3/14
		AGCCCTAGAG‐‐‐‐‐‐‐‐‐‐‐‐‐‐‐‐‐‐**(99)**‐‐‐‐‐‐‐‐ATGCGCAGACGCTGAAGCTTCTACC(27)CTACAGTGGAATCTGGTGAGATGGCAA	−125 bp 5/14
		AGCCCTAGAT‐‐‐‐‐‐‐‐‐‐‐‐‐‐AAGAT(99)CACCCTGGAT‐‐‐‐‐‐‐‐‐‐‐‐‐‐‐‐‐‐‐‐‐‐‐**(15)**‐‐‐‐‐‐‐‐‐‐‐‐‐‐‐‐‐‐AGATGGCAA	−14 bp/−56 bp 6/14
	G1P03	AGCCCTAGATGTCTGTCGATCCTCAAGAT(99)CACCCTGGATGCGCAGACGCTGAAGCTTCTACC(27)CTACAGTGGAATCTGGTGAGATGGCAA	WT 2/8
		AGCCCTAGAT‐‐‐‐‐‐‐‐‐‐‐‐‐‐‐‐‐‐‐**(99)**‐‐‐‐‐‐‐‐‐TGCGCAGACGCTGAAGCTTCTACC(27)CTACAGTGGAATCTGGTGAGATGGCAA	−127 bp 3/8
		AGCCCTAGATGTCTGTCGATCCTCAAGAT(99)CACCCTGGA‐‐‐‐‐‐‐‐‐‐‐‐‐‐‐‐‐‐‐‐‐‐‐‐**(15)**‐‐‐‐‐‐‐‐‐‐‐‐‐‐‐‐‐‐AGATGGCAA	−57 bp 3/8
	G1P04	AGCCCTAGATGTCTGTCGATCCTCAAGAT(99)CACCCTGGATGCGCAGACGCTGAAGCTTCTACC(27)CTACAGTGGAATCTGGTGAGATGGCAA	WT 1/5
		AGCCCTAGAG‐‐‐‐‐‐‐‐‐‐‐‐‐‐‐‐‐‐**(99)**‐‐‐‐‐‐‐‐ATGCGCAGACGCTGAAGCTTCTACC(27)CTACAGTGGAATCTGGTGAGATGGCAA	−125 bp 2/5
		AGCCCTAGAT‐‐‐‐‐‐‐‐‐‐‐‐‐‐AAGAT(99)CACCCTGGAT‐‐‐‐‐‐‐‐‐‐‐‐‐‐‐‐‐‐‐‐‐‐‐**(15)**‐‐‐‐‐‐‐‐‐‐‐‐‐‐‐‐‐‐AGATGGCAA	−14 bp/−56 bp 2/5

*Note*: The offsprings were generated by natural mating of 8 GE female pigs with previously generated 4 GE male pigs (GTKO/hCD55/hTBM/hCD39) [21].

Abbreviation: GE = gene edited.

**TABLE 8:** Summary of genotypes of F1 generation offspring of 8‐GEC pigs.

No.	Father	Mother	F1 generation	GGTA1 Exon 3	GGTA1 Exon 8	CMAH	β4GalNT2	hCD46	hCD55	hCD59	hTBM	hCD39
1	DN4TGF06P10P12			WT	+3 bp/△26 bp +1 bp/△3 bp	WT	WT	×	○	×	○	○
2		DN8TGC12F01P01		△52/△52	WT	△3/△124	△125/△14△56/ △127/△57	○	○	○	○	○
3			G1P01	WT/△52	WT/+3△26	WT/△3	WT/△125/△14△56	○	○	○	○	○
4			G1P02	WT/△52	WT/+3△26	WT/△3	WT/△125/△14△56	○	○	○	○	○
5			G1P03	WT/△52	WT/+3△26	WT/△3	WT/△127/△57	×	○	×	○	○
6			G1P04	WT/△52	WT/+3△26	WT/△124	WT/△125/△14△56	○	○	○	○	○

*Note*: ‘○’, insertion; ‘×’, no insertion. The offsprings were generated by natural mating of 8 GE female pigs with previously generated 4 GE male pigs (GTKO/hCD55/hTBM/hCD39) [21].

Abbreviation: GE = gene edited.

### 3.3 Kidney Xenotransplantation From 8GEC Pigs to Non‐human Primates

To evaluate the effectiveness of 8‐GEC pigs as donor pigs for xenotransplantation, we carried out two cases of pig‐to‐monkey kidney transplantation. First, we collected the serum from 7 monkeys and conducted a cross‐matching experiment with the PBMCs of two 8‐GC pigs (P03 and P06) and selected 2 monkeys (R24#, R25#) as recipients for kidney xenotransplantation (Figure S3). Two days before surgery, immunosuppression was performed with anti‐CD20 mAb and ATG, respectively. On the day of the operation, the P03 pig suddenly died during the process of obtaining the kidney, so we first performed a kidney transplant from the P06 pig to the R25# monkey. After removing one kidney of the monkey, the pig kidney was anastomosed to the monkey's blood vessel in situ and the blood flow was opened. The colour of the pig kidney immediately changed from pink white to bright red, and no HAR was found (Figure S3B). Considering that the pig kidney may be difficult to maintain function due to its small size, we retained a monkey kidney and completed the transplantation.

Next, we re‐selected 8‐GEC pigs with a suitable weight and continued to complete the kidney xenotransplantation from pig to monkey R24# (M1). Additionally, we added another rhesus monkey (M2) to conduct kidney xenotransplantation. Three days before the operation, immunosuppression was performed with anti‐CD20 mAb, ATG and CVF respectively. On the day of surgery, we first removed one monkey kidney, then transplanted the pig kidney to the recipient monkey and finally removed another monkey kidney. The transplantation operation was successful, and the porcine kidney produced urine within minutes after the transplantation, and no HAR was found. To facilitate postoperative drug administration, we performed a gastrostomy in the recipient monkeys. During the postoperative care process, regular administration was carried out according to the established immunosuppressive regimen (Figure S3A), and corresponding treatment or nutritional supplements were given accordingly. We monitored the concentrations of drugs administered through gastrostomy and found that tacrolimus, MMF and sirolimus immunosuppressants were not detected in the blood 3 days after surgery. Later, we detected drug concentrations in serum only after replacing oral immunosuppressants with injectable dosages (Figure S3C). The ultrasonography of the recipient monkey on day 6 after surgery showed that the pig kidneys still maintained full blood flow without intravascular thrombosis (Figure S3D). Unfortunately, two recipients, M1 and M2, died on day 17 and 15 post‐transplantation.

**FIGURE 7:**
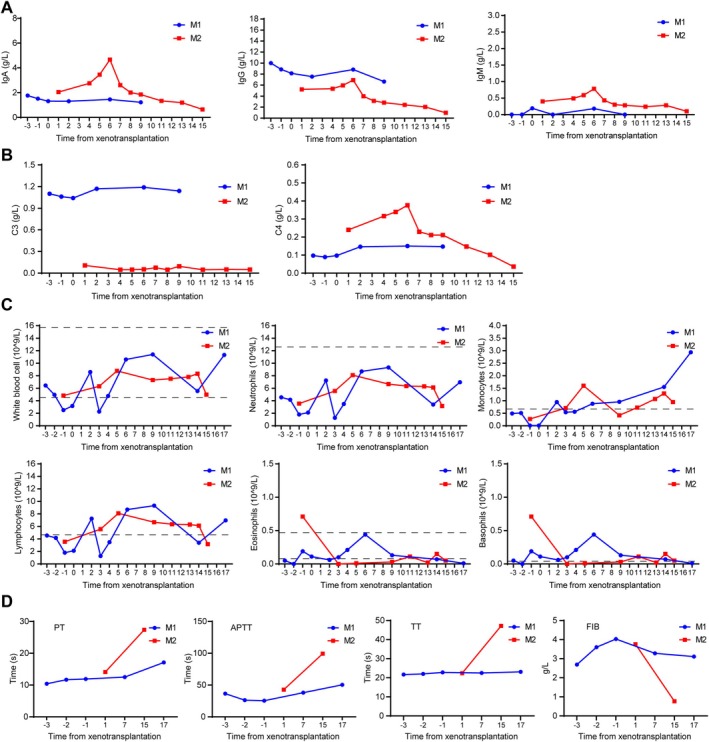
Humoral, cellular immunity, and coagulation of recipient monkey. (A) The levels of IgA, IgG, and IgM in the serum of the recipient monkey. (B) The levels of complement C3 and C4 in the serum of the recipient monkey. (C) The numbers of white blood cells(WBC), neutrophils, monocytes, lymphocytes, eosinophils, and basophils in the whole blood of the recipient monkey. (D) The coagulation indexes: Prothrombin time (PT), activated partial thromboplastin time (APTT), thrombin time (TT) and fibrinogen (FIB) in the recipient monkey.

We summarised the entire pig‐to‐monkey kidney transplantation process. In terms of immune rejection, the serum antibodies IgA, IgG and IgM, and complements C3 and C4 of the recipient monkeys were maintained at relatively stable levels (Figure 7A,B).

The number of leukocytes, neutrophils, lymphocytes, eosinophils and basophils was within a controllable range, while the number of monocytes gradually increased during the process (Figure 7C). In terms of coagulation, PT, APTT, TT and FIB remained relatively stable in the recipient M1 but increased significantly in the recipient M2 (Figure 7D).

**FIGURE 8:**
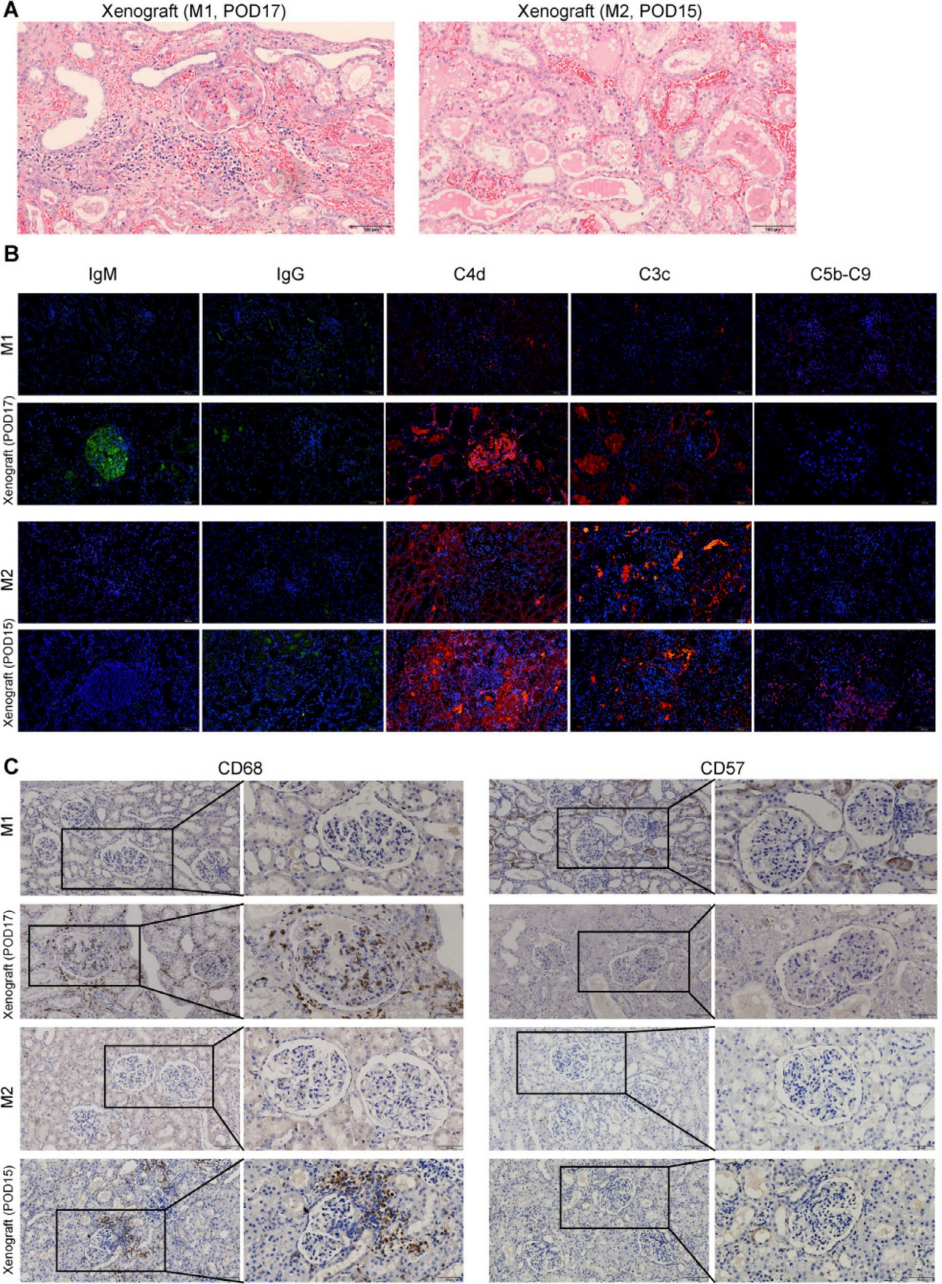
Pathological section of renal tissue. (A) HE staining of kidney xenograft. (B) Immunoglobulins and complement deposition in 8‐GE porcine kidney xenograft confirmed by immunofluorescence. (C) CD68^+^ macrophages and CD57^+^ NK cells infiltration in porcine kidney xenograft confirmed by immunohistochemistry; scale bar = 100 μm.

In terms of renal pathology, HE staining revealed that there was congestion in the lumen of capillary loops within the glomerulus and the peritubular capillaries, local interstitial bleeding, interstitial inflammatory cell infiltration, significant swelling of some renal tubular epithelial cells and disintegration of a few renal tubular epithelial cells (Figure 8A). Similarly, PAS and Masson staining showed that there was micro‐thrombotic embolism of glomerular capillaries, focal interstitial haemorrhage, swelling and disintegration of partial renal tubular epithelial cells and slight renal tubular inflammation (Figure S5). Immunofluorescence staining revealed the deposition of IgG and IgM antibodies as well as complement C4d, C3c and C5b‐C9 in the xenograft (Figure 8B). Immunohistochemical staining showed that the graft was infiltrated with CD68^+^ macrophages but without CD57^+^ NK cells (Figure 8C). In addition, sporadic CD3/CD8 positive T cells were found in the xenograft by immunofluorescence (Figure S6). Based on the Banff lesion scoring of renal allograft pathology, recipients M1 and M2 mainly developed active antibody‐mediated rejection (Table 9). The above results suggested that 8GE pig kidneys can effectively overcome the HAR but might develop acute humoral rejection and cellular immune rejection.

**TABLE 9:** Banff lesion scoring at necropsy.

Animal ID	ATI	TMA	g	i	t	v	ptc	cg	ci	ct	cv	mm	i‐IFTA	ti	c4d	Rejection phenotype allotransplantation‐banff
M1 (100229)	Yes	Yes	1	1	0	0	0	0	1	0	2	1	1	1	1	aAMR
M2 (11065)	Yes	Yes	1	1	1	0	0	0	1	0	1	1	1	0	2	CAMR

Abbreviations: aAMR, active antibody mediated rejection; CAMR, chronically active antibody mediated rejection; cg, glomerular basement membrane double contours; ci, interstitial fibrosis; ct, tubular atrophy; cv, vascular fibrous intimal thickening; g, glomerulitis; i, interstitial inflammation; i‐IFTA, inflammation in area of IFTA; mm, mesangial matrix expansion; ptc, peritubular capillaritis; t, tubulitis; ti, total inflammation; v, intimal arteritis.

**FIGURE 9:**
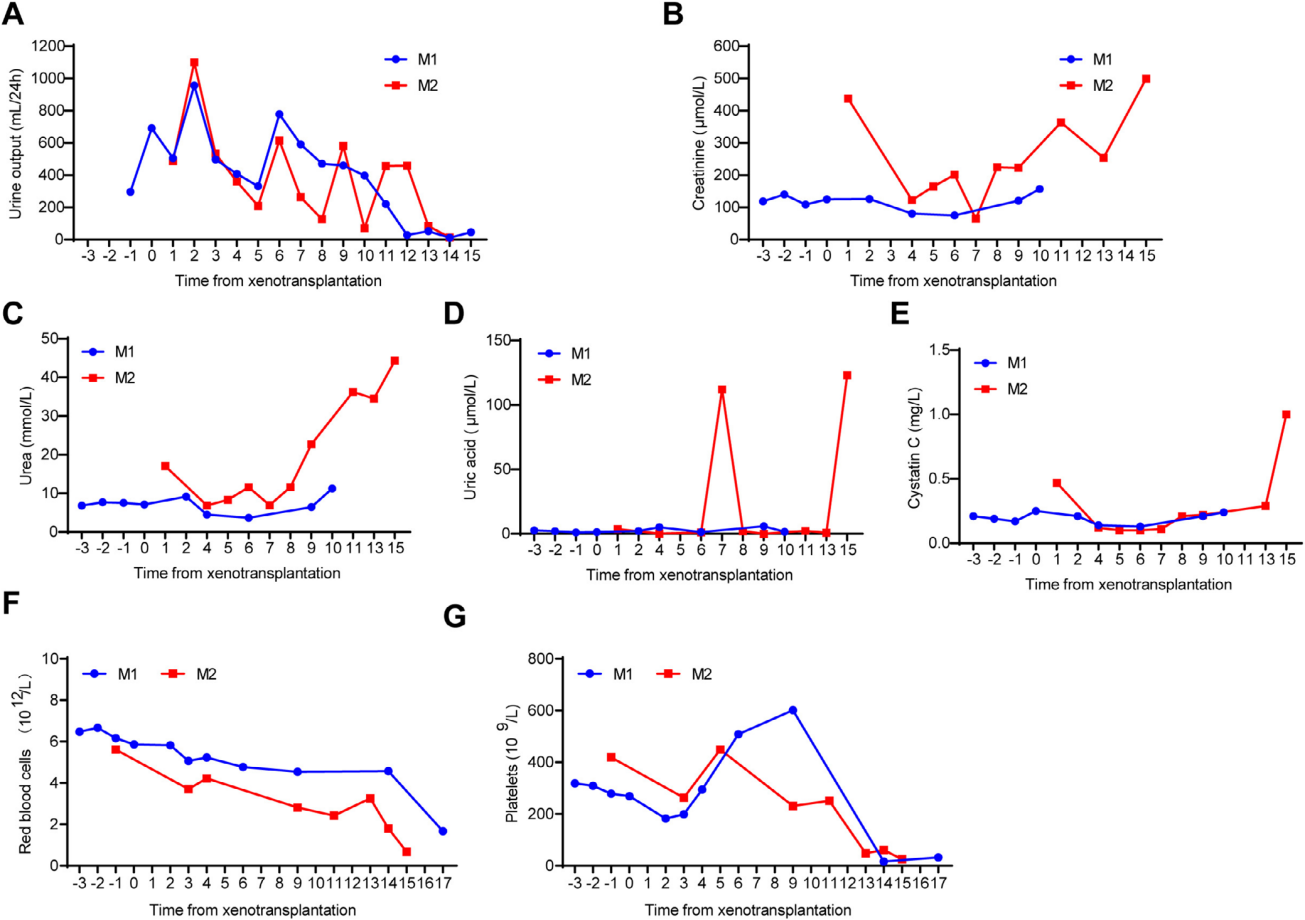
The renal function and erythrocyte indexes of the recipient monkey. (A) 24‐h urine output. (B–E) The levels of creatinine (B), urea (C), uric acid (D) and cystatin C (D) in the serum of recipient monkeys. (F–G) The numbers of platelets (F) and red blood cells (G).

In terms of renal function, the 24‐h urine output of recipient monkeys remained well in the early stages of post‐transplantation but gradually decreases in the later stages of post‐transplantation (Figure 9A). The serum creatinine levels remained stable in the recipient M1 but fluctuated in the recipient M2 (Figure 9B). The serum urea level also remained stable in the recipient M1 but was obviously elevated in the recipient M2 (Figure 9C). Meanwhile, the other indicators such as serum uric acid and cystatin C were maintained at relatively stable levels (Figure 9D,E), indicating that the kidney function of the 8‐GE pigs was basically normal. The kidney also promoted the production of RBCs and therefore, we monitored the changes in the number of RBCs. It was found that RBCs showed a gradual decreasing trend (Figure 9F). In addition, both recipients developed severe thrombocytopenia (Figure 9G).

In terms of liver and other physiological functions, various liver function indicators, including TP, ALB, GLB, TBIL, TBA, AST, ALT, ADA and ALP, remained relatively stable. Electrolytes Na, Cl, HCO_3_, Mg, P, Ca and K also remained stable (Figure S4).

## 4 Discussion

Pig‐to‐human xenotransplantation is about to enter the clinical phase; thus, it is essential to construct and select donor pigs with the most suitable genetic combination. The number of multigene‐modified donor pigs for xenotransplantation is increasing with the advent of gene editing technologies. However, which gene combination is suitable for organ‐ or tissue‐specific transplantation remains unclear, and as the number of gene edits increases, it becomes more difficult and more time‐consuming to obtain viable donor pigs. In this study, we used the CRISPR/Cas9 system, PiggyBac transposase system and somatic cell cloning technology to obtain 8‐GEC pigs and verified their effectiveness by transplanting the 8GE kidney to NHP, thereby providing clinical evidence for pig‐to‐human kidney transplantation and laying down the foundation for xenotransplantation research.

In order to produce 8GEC pigs, we integrated 7 sgRNAs into one plasmid to achieve simultaneous KO of multiple porcine genes. This method is more effective than co‐transfection of 7 plasmids, as simultaneous transfer of multiple plasmids is difficult and highly cytotoxic to cells. Recent research has also shown that this method can achieve efficient simultaneous editing of multiple genes [30]. Multiple sgRNAs might increase off‐target risk, but our 8‐GEC pigs carried only 3 off‐targets among 1042 off‐target candidates (Table S4). Two of them were located in the intergenic region between chromosomes 1 and 14, while the 3rd one on the LOC110255214 gene on chromosome 12. We analysed a total of 6 cloned individuals from the same donor cell line, while the off‐target on chromosome 1 was detected in 4 individuals, and the one on chromosome 14 was detected in 3 individuals, suggesting that these two off‐targets may not be caused by the CRISPR‐Cas9 system. The LOC110255214 gene showed off‐target effects in all six individuals, which is similar to the β4GalNT2 gene and may also be a potential xeno‐antigen. Overall, the off‐target effects caused by the CRISPR‐Cas9 system are minimal, consistent with off‐target situations in previous GEC pigs obtained through CRISPR‐Cas9 and somatic cell cloning [31].

Among eight randomly selected cell colonies, the editing efficiency of the GGTA1 gene was approximately 100%. On the premise of GTKO, the colonies with KO of CMAH and β4GalNT2 were further identified, and finally, a cell line with 13 genomic edits was selected. However, there were three genotypes of the GGTA1 gene in this cell line, which may be due to impure cell colonies. Therefore, we obtained the fetus through the first round of somatic cell cloning and conducted genotyping, gene expression identification, and karyotyping analysis to determine that it was a normal fetus with 8GE. We then recloned to obtain an 8‐GEC pig, and the proportion of healthy piglets among the cloned pigs was as high as 82% (23/28) after recloning. This once again proved that the method of first cloning to obtain foetuses, identifying the foetuses, and then re‐cloning to obtain the piglets has effectively improved the production efficiency of GEC pigs [32]. In addition, the integrated sequence length for plasmids of hCD46, hCD55, hCD59, hTBM, hCD39 and promoters reached up to 15 kb, and the piggyBac transposons system was used for transgenes to achieve efficient large fragment integration (64%, 16/25), which is similar to our previous study [33]. However, the PiggyBac transposon system randomly integrates exogenous genes into the pig genome accompanied by multiple copies, which might disrupt functional genes and cause by‐effects when an organ is transplanted into recipients. Fortunately, three transgenic copies were integrated into the introns of the pig RFTN1 and MYO10 genes, as well as the intergenic region between the PRLR and LOC110257300 genes, thus it is intriguingly speculated that they have little by‐effects.

In the 8‐GEC pigs, the expression of hCD46, hCD55, hCD59, hTBM and hCD39 in different tissues was detected. However, the expression levels of each gene were not consistent among different individuals cloned from the same fetal cell line, which could be due to reprogramming during cloning. Moreover, we found that genes with high mRNA expression levels were not equally expressed at the protein level. For example, hCD59 has low mRNA levels in porcine kidneys, while protein levels were higher (Figure 4). Moreover, the expression of the same gene (hCD59) is also different in various tissues of the same cloned pig (Figure S1) and there are clearly visible double peaks (negative, and positive peaks) in the flow cytometry results for hCD59 expression (Figure 3G and Figure 6C), which may be due to different degrees of epigenetic modification of the human genes in the pig genome [21, 34]. Nevertheless, strategies such as codon optimization, site‐specific knock‐in and usage of porcine endogenous promoter, etc., might be more beneficial for consistent transgene expression. Despite this, the pig kidneys removed from the recipient monkey after loss of function still exhibited high expressions of all human genes hCD46, hCD55, hCD59, hTBM and hCD39 as shown by immunofluorescence staining (Figure S3E).

In pig‐to‐monkey kidney transplantation, the 8‐GE pig kidney showed good anti‐immune rejection effects. First, in vitro cross‐matching experiments revealed that PBMCs from 8‐GEC pigs had significantly reduced monkey IgG and IgM binding abilities and increased anti‐complement‐dependent cytotoxicity. Secondly, during the 9 days post‐operative observation, the immunoglobulins IgG, IgM and IgA, as well as complements C3 and C4, remained relatively stable in the recipient monkeys, suggesting that the transplantation of the kidney xenograft with TKO (GGTA1, CMAH, β4GalNT2), along with the expression of three complement regulatory proteins (CD46, CD55, CD59) effectively protected against HAR. However, at the experimental end point, the number of monocytes in the recipient monkeys increased significantly. Immunohistochemical analysis revealed obvious infiltrates of CD68^+^ macrophages in the pig kidney grafts, suggesting that acute rejection occurred later. However, it has been reported that acute rejection is reversible, and timely adjustment of the dosage of immunosuppressants can help recipient monkeys to overcome the risk period of acute rejection [35]. Additionally, on the basis of 8‐GE pigs, further expression of hCD47 can also effectively inhibit acute rejection mediated by NK cells and macrophages [36]. In terms of anticoagulation, PT, TT and FIB remained relatively stable, and only APTT showed an increasing trend in the late transplantation period, indicating that hTBM and hCD39 overexpression effectively controlled coagulation. However, near the end of the experiment, the number of platelets dropped sharply, which may be related to the use of anti‐inflammatory drugs [37].

Within 10 days after surgery, the 8GE pig kidney functioned normally. The recipient monkey's serum creatinine, cystatin C, uric acid and urea nitrogen were maintained at relatively stable levels, and the 24‐h urine output was above 400 mL. In addition, immunosuppressants, antibiotics and other drugs did not cause any obvious liver damage or electrolyte imbalance. Unfortunately, an autopsy revealed that the recipient monkey had gastric perforation, and food and medicines leaked into the abdominal cavity. The immunosuppressant blood concentration of the recipient monkey did not meet the standard levels (Figure S3C), and also severe nutritional deficiencies might affect the survival time of the recipient monkey. In addition, we had performed the liver and kidney co‐transplantation from our 8‐GEC pigs to brain‐dead humans, which functioned normally without HAR during a 7‐day observation time. Thus, we believe that our 8‐GEC pigs can achieve long‐term survival upon improvements in postoperative care plans and experiences. However, it is still difficult to define the genetic combination more suitable for kidney, heart or liver xenotransplantation, as the heart xenograft from 10 GE pigs transplanted into 2 live human patients even exhibited different survival times of 42 and 60 days [1, 3]. Besides the genetic modification of donor pigs, other factors such as immunosuppressive regimens and cross‐species infections are also critical for the survival of xenografts. In terms of cross‐species infections, like porcine endogenous retroviruses (PERVs), it can infect human cells and integrate its own genome into the human genome [38]. Once integration occurs, the human genome will be disrupted, potentially leading to serious health problems such as tumours and immune deficiencies [39]. It is even possible that PERV spread among populations, causing serious public health issues similar to the HIV virus. The World Health Organisation has specifically identified PERV infection as a concern that must be considered in the clinical application of xenotransplantation [40]. Nevertheless, the inactivation of PERV using CRISPR‐Cas9 [41] can effectively mitigate its transmissions, but the risk for the transmission of infection due to novel pathogens in association with xenotransplantation is unknown. Therefore, we believe that the clinical application of GE pigs for xenotransplantation should eliminate the transmissions of zoonotic pathogens in the future.

In summary, this study used the CRISPR/Cas9 system, PiggyBac transposase system, and somatic cell cloning technology to successfully obtain 8GEC donor pigs and proved that 8 gene editing effectively alleviates the immune incompatibility in xenografts, as evidenced by the survival of the kidney xenograft from these pigs to NHP.

## Author Contributions

H.‐J.W. conceived and designed the experiment; K.D., H.‐Y.Z. and Z.Z. designed the research. J.W., K.X., H.Z., M.A.J., X.H., C.Y., H.G., F.W., and G.C. performed the molecular experiments and analysed data. D.J. and H.Z. transfected the cells and performed a cell culture. J.G., T.W., K.L. and X.Z. carried out SCNT and embryo transfer. T.L., G.C., H.H., S.Q., G.W., H.‐J.W., and Z.Z. performed pig‐to‐monkey kidney xenotransplantation. K.X. and M.A.J. wrote the manuscript. H.‐J.W., H.‐Y.Z., Z.Z., K.D., K.X. and M.A.J. revised the manuscript and validated the data. All authors contributed to the article and approved the submitted version.

## Conflicts of Interest

The authors declare no conflicts of interest.

## Supporting information


**Figure S1.** Immunofluorescence staining of different tissues of 8‐GEC piglets. The protein expression of αGal, Neu5Gc, Sda, hCD46, hCD55, hCD59, hTBM and hCD39 genes in kidney, lung, liver, spleen and heart of cloned piglets was confirmed by immunofluorescence.

**Figure S2.** Analysis of transgenic insertion in 5 other 8‐GEC pigs by WGS.

**Figure S3.** Immunosuppressive regimen and other observations (A) Immunosuppressive regimen. (B) Changes of pig kidney before and after opening the blood vessel during transplantation into rhesus monkeys (C) Measurement of immunosuppressant concentrations in the blood. (D) Doppler ultrasonography of pig kidney xenograft after 6 days of surgery. (E) Expressions human genes after kidney function loss as showen by immunofluorescence staining.

**Figure S4.** The liver functions indexes and electrolytes of recipient monkey. Liver function indexes: total protein (TP), ailbumin (ALB), gloubulin (GLB), total bilirubin (TBIL), total bile acid (TBA), aspartate transaminase (AST), alanine transaminase (ALT), adenosine deaminase (ADA) and alkaline phosphatase (ALP); Electrolytes: sodium (Na), chlorine (Cl), bicarbonate (HCO3), calcium (Ca), magnesium (Mg), phosphorus (P) and potassium (K).

**Figure S5.** Masson and Pas staining of kidney xenograft (scale bar = 200 μm).

**Figure S6.** T cell infiltration in porcine kidney xenograft confirmed by immunofluorescence (scale bar = 100 μm).

**Table S1.** Primers for genotyping, qPCR and ddPCR.

**Table S2.** List of antibodies used for protein expression.

**Table S3.** Off‐target candidate sites predicted by Cas‐OFFinder on whole genome of 8‐GEC pigs.

**Table S4.** Summary of off‐target of 8‐GEC pigs.

**Table S5.** Annotation information of transgenic insertion location.

## Data Availability

The data generated in the present study may be shared on reasonable request from the corresponding author. The raw sequencing data generated in this study can be accessed on the Genome Sequence Archive (GSA) under the accession CRA024012 (https://ngdc.cncb.ac.cn/gsa/browse/CRA024012).

## References

[1] B. P. Griffith and C. E. Goerlich , “Genetically Modified Porcine‐To‐Human Cardiac Xenotransplantation,” New England Journal of Medicine 387, no. 1 (2022): 35–44, 10.1056/NEJMoa2201422. 35731912 PMC10361070

[2] N. Moazami , J. M. Stern , K. Khalil , et al., “Pig‐To‐Human Heart Xenotransplantation in Two Recently Deceased Human Recipients,” Nature Medicine 29, no. 8 (2023): 1989–1997. 10.1038/s41591-023-02471-9 37488288

[3] M. Vadori and E. Cozzi , “Current Challenges in Xenotransplantation,” Current Opinion in Organ Transplantation 29, no. 3 (2024): 205–211. 38529696 10.1097/MOT.0000000000001146 PMC11064916

[4] B. M. Kuehn , “Pig‐To‐Human Xenotransplants Take Another Step Forward,” Kidney News 15, no. 10 (2023): 7–8.

[5] R. A. Montgomery , J. M. Stern , B. E. Lonze , et al., “Results of Two Cases of Pig‐To‐Human Kidney Xenotransplantation,” New England Journal of Medicine 386, no. 20 (2022): 1889–1898. 35584156 10.1056/NEJMoa2120238

[6] P. M. Porrett , B. J. Orandi , V. Kumar , et al., “First Clinical‐Grade Porcine Kidney Xenotransplant Using a Human Decedent Model,” American Journal of Transplantation: Official Journal of the American Society of Transplantation and the American Society of Transplant Surgeons 22, no. 4 (2022): 1037–1053, 10.1111/ajt.16930. 35049121

[7] J. E. Locke , V. Kumar , D. Anderson , and P. M. Porrett , “Normal Graft Function After Pig‐To‐Human Kidney Xenotransplant,” JAMA Surgery 158, no. 10 (2023): 1106–1108. 37585176 10.1001/jamasurg.2023.2774 PMC10433134

[8] Y. Wang , G. Chen , D. Pan , et al., “Pig‐To‐Human Kidney Xenotransplants Using Genetically Modified Minipigs,” Cell Reports Medicine 5, no. 10 (2024): 101744. 39317190 10.1016/j.xcrm.2024.101744 PMC11513830

[9] L. Bernstein , “Pig Kidney Transplant in Brain‐Dead Man Marks Advance, NYU Surgeons Say,” Washington Post (2023), https://www.washingtonpost.com/health/2023/08/16/pig‐kidney‐transplant‐nyu.

[10] S. Mallapaty and M. Kozlov , “First Pig Kidney Transplant in a Person: What It Means for the Future,” Nature 628, no. 8006 (2024): 13–14. 38519547 10.1038/d41586-024-00879-y

[11] M. Langin , T. Mayr , B. Reichart , et al., “Consistent Success in Life‐Supporting Porcine Cardiac Xenotransplantation,” Nature 564, no. 7736 (2018): 430–433. 30518863 10.1038/s41586-018-0765-z

[12] A. K. Singh , C. E. Goerlich , T. Zhang , et al., “Genetically Engineered Pig Heart Transplantation in Non‐Human Primates,” Communications Medicine 5, no. 1 (2025): 6. 39774817 10.1038/s43856-025-00731-y PMC11707197

[13] S. C. Kim , D. V. Mathews , C. P. Breeden , et al., “Long‐Term Survival of Pig‐To‐Rhesus Macaque Renal Xenografts Is Dependent on CD4 T Cell Depletion,” American Journal of Transplantation: Official Journal of the American Society of Transplantation and the American Society of Transplant Surgeons 19, no. 8 (2019): 2174–2185, 10.1111/ajt.15329. 30821922 PMC6658347

[14] R. P. Anand , J. V. Layer , D. Heja , et al., “Design and Testing of a Humanized Porcine Donor for Xenotransplantation,” Nature 622, no. 7982 (2023): 393–401. 37821590 10.1038/s41586-023-06594-4 PMC10567564

[15] Q. Li and P. Lan , “Activation of Immune Signals During Organ Transplantation,” Signal Transduction and Targeted Therapy 8, no. 1 (2023): 110. 36906586 10.1038/s41392-023-01377-9 PMC10008588

[16] H. Hara , T. Yamamoto , H.‐J. Wei , and D. K. Cooper , “What Have We Learned From In Vitro Studies About Pig‐To‐Primate Organ Transplantation?,” Transplantation 107, no. 6 (2023): 1265–1277. 36536507 10.1097/TP.0000000000004458 PMC10205677

[17] P. J. Cowan and S. C. Robson , “Progress Towards Overcoming Coagulopathy and Hemostatic Dysfunction Associated With Xenotransplantation,” International Journal of Surgery 23 (2015): 296–300. 26220018 10.1016/j.ijsu.2015.07.682

[18] K. R. McCurry , D. L. Kooyman , C. G. Alvarado , et al., “Human Complement Regulatory Proteins Protect Swine‐To‐Primate Cardiac Xenografts From Humoral Injury,” Nature Medicine 1, no. 5 (1995): 423–427, 10.1038/nm0595-423. 7585088

[19] R. N. Pierson, 3rd , A. Dorling , D. Ayares , et al., “Current Status of Xenotransplantation and Prospects for Clinical Application,” Xenotransplantation 16, no. 5 (2009): 263–280, 10.1111/j.1399-3089.2009.00534.x. 19796067 PMC2866107

[20] M. J. Whitley , J. Suwanpradid , C. Lai , et al., “ENTPD1 (CD39) Expression Inhibits UVR‐Induced DNA Damage Repair Through Purinergic Signaling and Is Associated With Metastasis in Human Cutaneous Squamous Cell Carcinoma,” Journal of Investigative Dermatology 141, no. 10 (2021): 2509–2520. 33848530 10.1016/j.jid.2021.02.753

[21] C. Yang , Y. Wei , X. Li , et al., “Production of Four‐Gene (GTKO/hCD55/hTBM/hCD39)‐Edited Donor Pigs and Kidney Xenotransplantation,” Xenotransplantation 31, no. 4 (2024): e12881. 39185796 10.1111/xen.12881

[22] H. Wei , Y. Qing , W. Pan , et al., “Comparison of the Efficiency of Banna Miniature Inbred Pig Somatic Cell Nuclear Transfer Among Different Donor Cells,” PLoS One 8, no. 2 (2013): e57728. 23469059 10.1371/journal.pone.0057728 PMC3585185

[23] S. Fisher , A. Barry , J. Abreu , et al., “A Scalable, Fully Automated Process for Construction of Sequence‐Ready Human Exome Targeted Capture Libraries,” Genome Biology 12, no. 1 (2011): R1. 21205303 10.1186/gb-2011-12-1-r1 PMC3091298

[24] D. A. Wheeler , M. Srinivasan , M. Egholm , et al., “The Complete Genome of an Individual by Massively Parallel DNA Sequencing,” Nature 452, no. 7189 (2008): 872–876. 18421352 10.1038/nature06884

[25] S. Chen , Y. Zhou , Y. Chen , and J. Gu , “Fastp: An Ultra‐Fast All‐In‐One FASTQ Preprocessor,” Bioinformatics (Oxford, England) 34, no. 17 (2018): i884–i890. 30423086 10.1093/bioinformatics/bty560 PMC6129281

[26] V. Md , S. Misra , H. Li , and S. Aluru , “Efficient Architecture‐Aware Acceleration of BWA‐MEM for Multicore Systems,” 2019, In 2019 IEEE International Parallel and Distributed Processing Symposium (IPDPS).

[27] A. McKenna , M. Hanna , E. Banks , et al., “The Genome Analysis Toolkit: A MapReduce Framework for Analyzing Next‐Generation DNA Sequencing Data,” Genome Research 20, no. 9 (2010): 1297–1303. 20644199 10.1101/gr.107524.110 PMC2928508

[28] S. Bae , J. Park , and J. S. Kim , “Cas‐OFFinder: A Fast and Versatile Algorithm That Searches for Potential Off‐Target Sites of Cas9 RNA‐Guided Endonucleases,” Bioinformatics (Oxford, England) 30, no. 10 (2014): 1473–1475. 24463181 10.1093/bioinformatics/btu048 PMC4016707

[29] Z. Ivics and Z. Izsvák , “Transposons for Gene Therapy!,” Current Gene Therapy 6, no. 5 (2006): 593–607. 17073604 10.2174/156652306778520647

[30] J. Q. Zhang , J. X. Guo , X. J. Wu , et al., “Optimization of sgRNA Expression Strategy to Generate Multiplex Gene‐Edited Pigs,” Zoological Research 43, no. 6 (2022): 1005–1008. 36257831 10.24272/j.issn.2095-8137.2022.244 PMC9700489

[31] Z. Li , J. Lan , X. Shi , et al., “Whole‐Genome Sequencing Reveals Rare off‐Target Mutations in MC1R‐Edited Pigs Generated by Using CRISPR‐Cas9 and Somatic Cell Nuclear Transfer,” CRISPR Journal 7, no. 1 (2024): 29–40. 38353621 10.1089/crispr.2023.0034

[32] H. Zhao , Y. Li , T. Wiriyahdamrong , et al., “Improved Production of GTKO/hCD55/hCD59 Triple‐Gene‐Modified Diannan Miniature Pigs for Xenotransplantation by Recloning,” Transgenic Research 29, no. 3 (2020): 369–379. 32358721 10.1007/s11248-020-00201-2

[33] Y. Yue , W. Xu , Y. Kan , et al., “Extensive Germline Genome Engineering in Pigs,” Nature Biomedical Engineering 5, no. 2 (2021): 134–143. 10.1038/s41551-020-00613-9 32958897

[34] S. Y. Alhaji , S. C. Ngai , and S. Abdullah , “Silencing of Transgene Expression in Mammalian Cells by DNA Methylation and Histone Modifications in Gene Therapy Perspective,” Biotechnology & Genetic Engineering Reviews 35, no. 1 (2019): 1–25. 30514178 10.1080/02648725.2018.1551594

[35] J. E. Cooper , “Evaluation and Treatment of Acute Rejection in Kidney Allografts,” Clinical Journal of the American Society of Nephrology 15, no. 3 (2020): 430–438. 32066593 10.2215/CJN.11991019 PMC7057293

[36] S. Nomura , Y. Ariyoshi , H. Watanabe , et al., “Transgenic Expression of Human CD47 Reduces Phagocytosis of Porcine Endothelial Cells and Podocytes by Baboon and Human Macrophages,” Xenotransplantation 27, no. 1 (2020): e12549. 31495971 10.1111/xen.12549 PMC7007337

[37] M. H. Bikhet , C. Hansen‐Estruch , M. Javed , et al., “Profound Thrombocytopenia Associated With Administration of Multiple Anti‐Inflammatory Agents in Baboons,” Immunity, Inflammation and Disease 10, no. 3 (2022): e588. 35049144 10.1002/iid3.588 PMC8926498

[38] U. Martin , V. Kiessig , J. H. Blusch , et al., “Expression of Pig Endogenous Retrovirus by Primary Porcine Endothelial Cells and Infection of Human Cells,” Lancet (London, England) 352, no. 9129 (1998): 692–694, 10.1016/S0140-6736(98)07144-X. 9728985

[39] J. Denner , “Porcine Endogenous Retroviruses in Xenotransplantation,” Nephrology, Dialysis, Transplantation: Official Publication of the European Dialysis and Transplant Association—European Renal Association 39, no. 8 (2024): 1221–1227. 38281060 10.1093/ndt/gfae023

[40] J. A. Fishman , L. Scobie , and Y. Takeuchi , “Xenotransplantation‐Associated Infectious Risk: A WHO Consultation,” Xenotransplantation 19, no. 2 (2012): 72–81. 22497509 10.1111/j.1399-3089.2012.00693.x PMC3768267

[41] D. Niu , H. J. Wei , L. Lin , et al., “Inactivation of Porcine Endogenous Retrovirus in Pigs Using CRISPR‐Cas9,” Science 357, no. 6357 (2017): 1303–1307. 28798043 10.1126/science.aan4187 PMC5813284

