# Supplementary figures and images for: Production and Functional Verification of 8‐Gene (GGTA1, CMAH, β4GalNT2, hCD46, hCD55, hCD59, hTBM, hCD39)‐Edited Donor Pigs for Xenotransplantation
Source: Cell Prolif. 2025 Apr 6;58(9):e70028. doi: 10.1111/cpr.70028 (PMC12414638; doi:10.1111/cpr.70028)

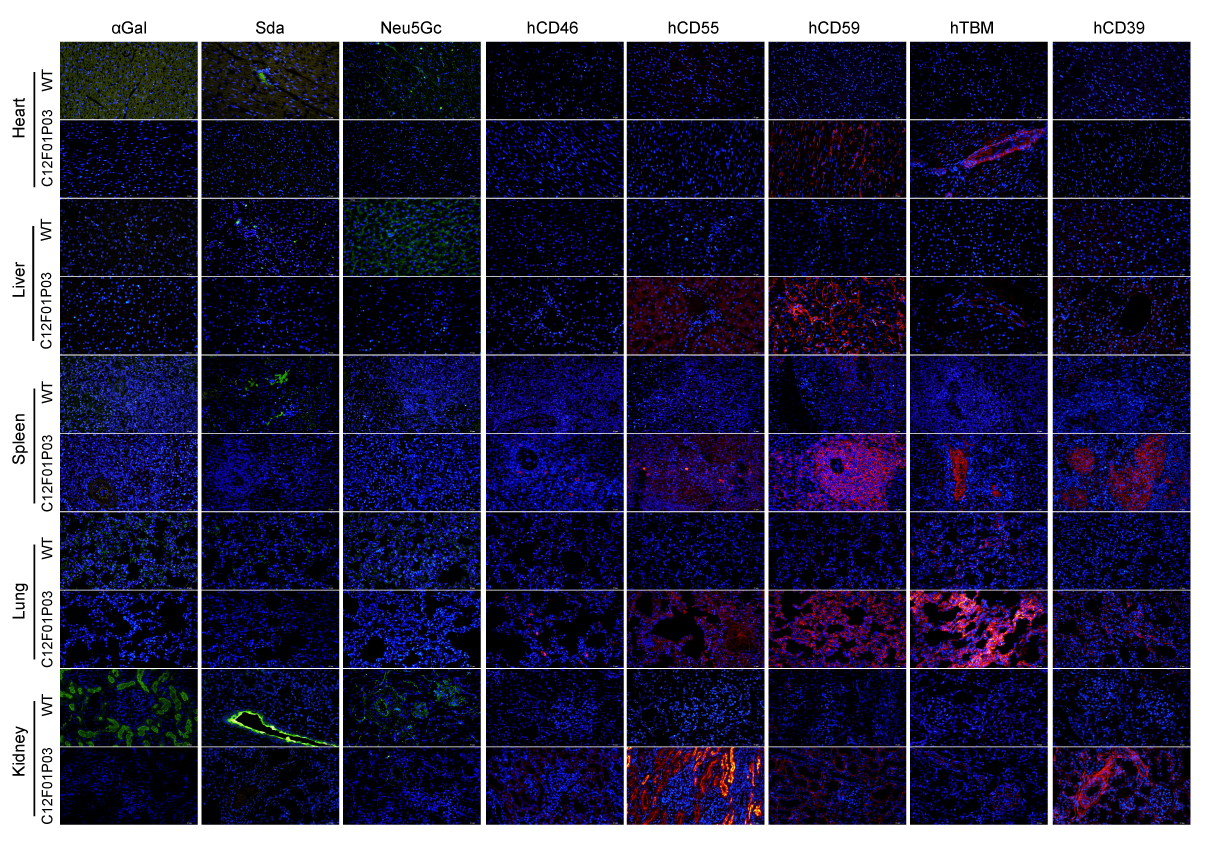

Supplement: Supplementary file 1 — Figure S1. Immunofluorescence staining of different tissues of 8‐GEC piglets. The protein expression of αGal, Neu5Gc, Sda, hCD46, hCD55, hCD59, hTBM and hCD39 genes in kidney, lung, liver, spleen and heart of cloned piglets was confirmed by immunofluorescence. [file CPR-58-e70028-s006.tif]

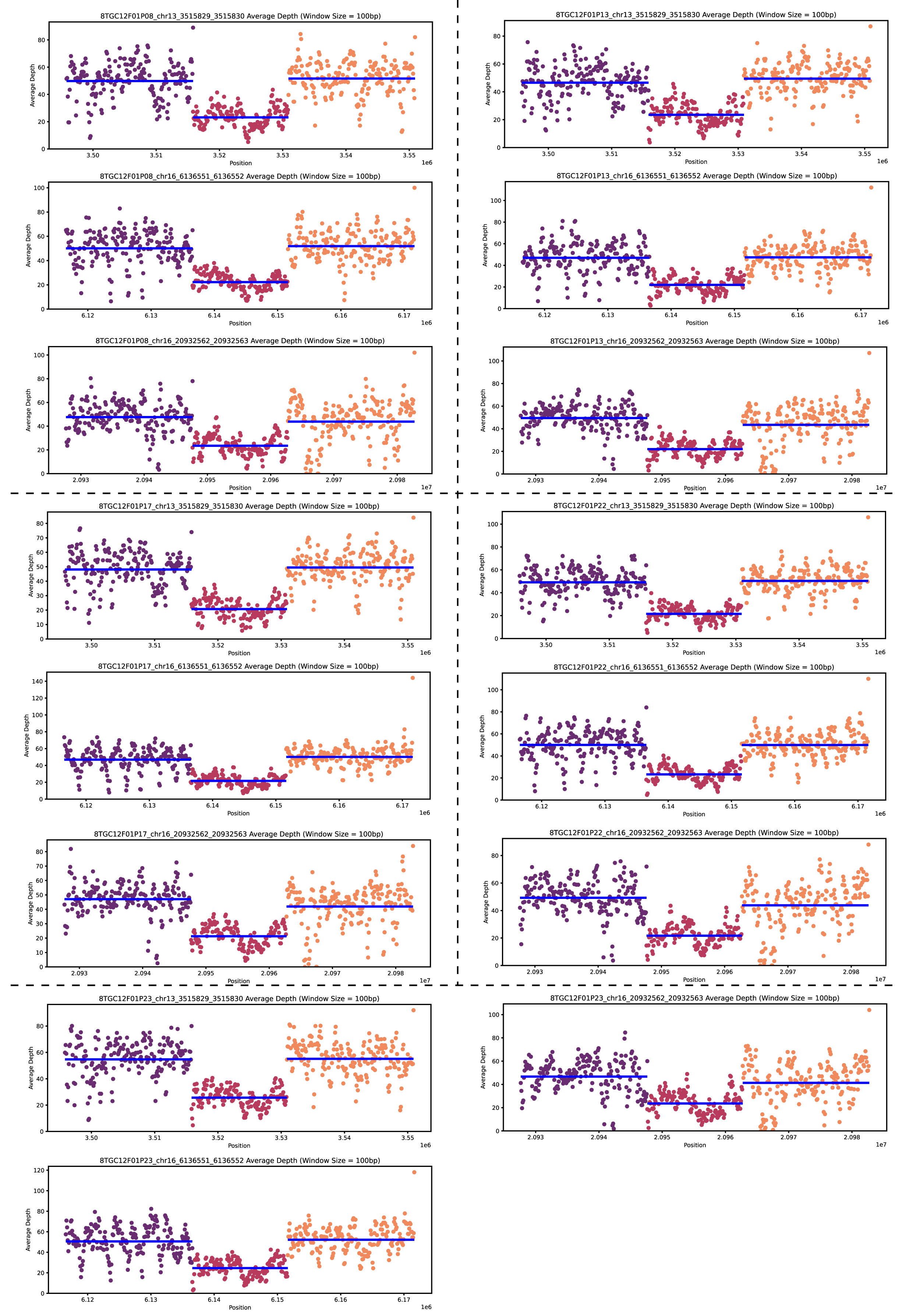

Supplement: Supplementary file 2 — Figure S2. Analysis of transgenic insertion in 5 other 8‐GEC pigs by WGS. [file CPR-58-e70028-s003.tif]

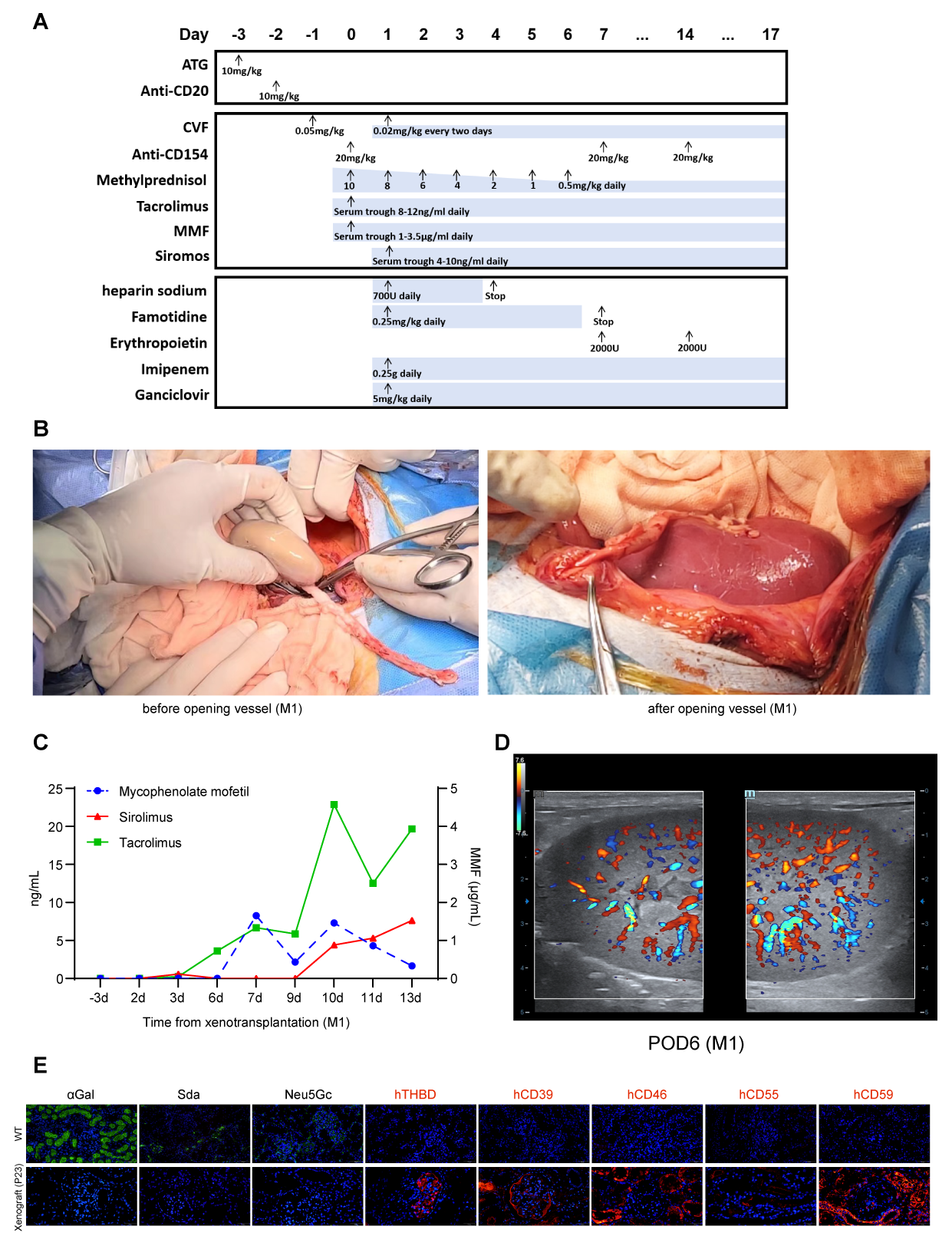

Supplement: Supplementary file 3 — Figure S3. Immunosuppressive regimen and other observations (A) Immunosuppressive regimen. (B) Changes of pig kidney before and after opening the blood vessel during transplantation into rhesus monkeys (C) Measurement of immunosuppressant concentrations in the blood. (D) Doppler ultrasonography of pig kidney xenograft after 6 days of surgery. (E) Expressions human genes after kidney function loss as showen by immunofluorescence staining. [file CPR-58-e70028-s002.tif]

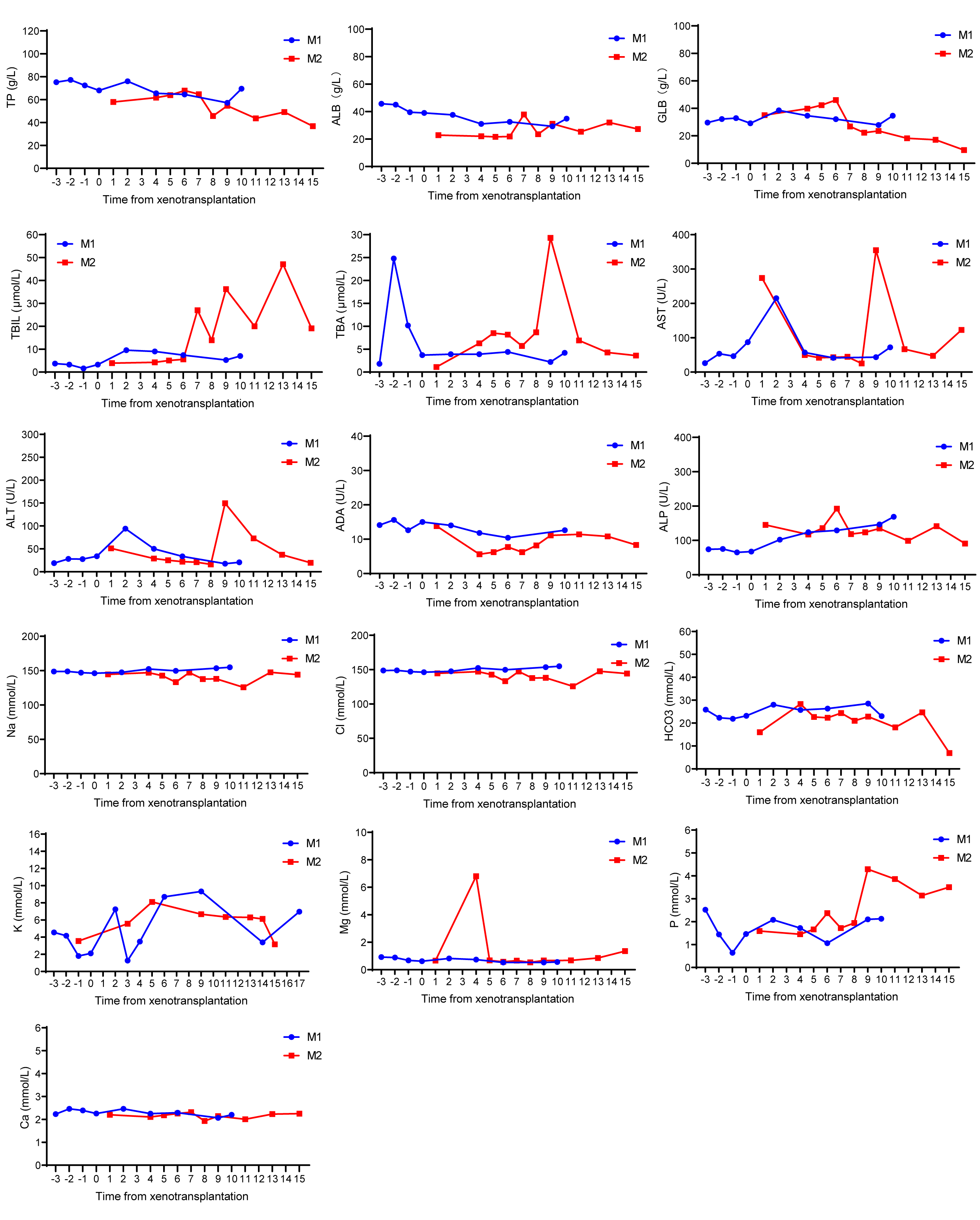

Supplement: Supplementary file 4 — Figure S4. The liver functions indexes and electrolytes of recipient monkey. Liver function indexes: total protein (TP), ailbumin (ALB), gloubulin (GLB), total bilirubin (TBIL), total bile acid (TBA), aspartate transaminase (AST), alanine transaminase (ALT), adenosine deaminase (ADA) and alkaline phosphatase (ALP); Electrolytes: sodium (Na), chlorine (Cl), bicarbonate (HCO3), calcium (Ca), magnesium (Mg), phosphorus (P) and potassium (K). [file CPR-58-e70028-s010.tif]

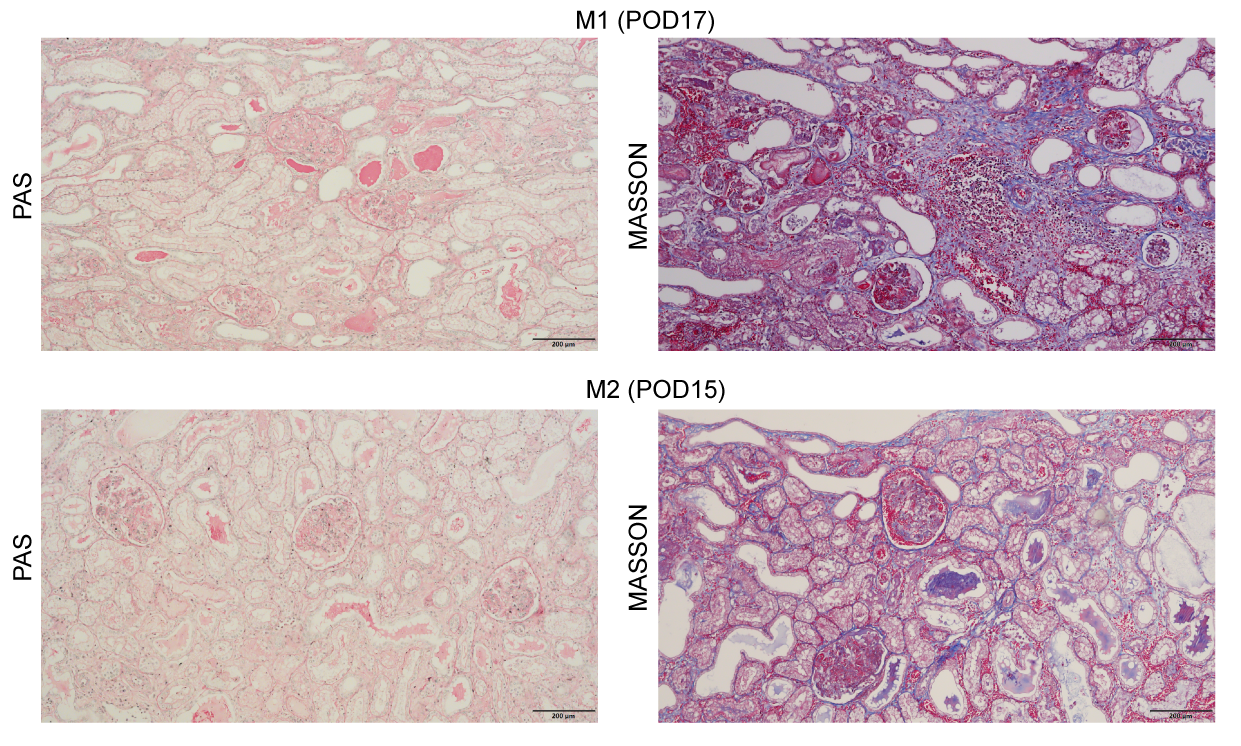

Supplement: Supplementary file 5 — Figure S5. Masson and Pas staining of kidney xenograft (scale bar = 200 μm). [file CPR-58-e70028-s001.tif]

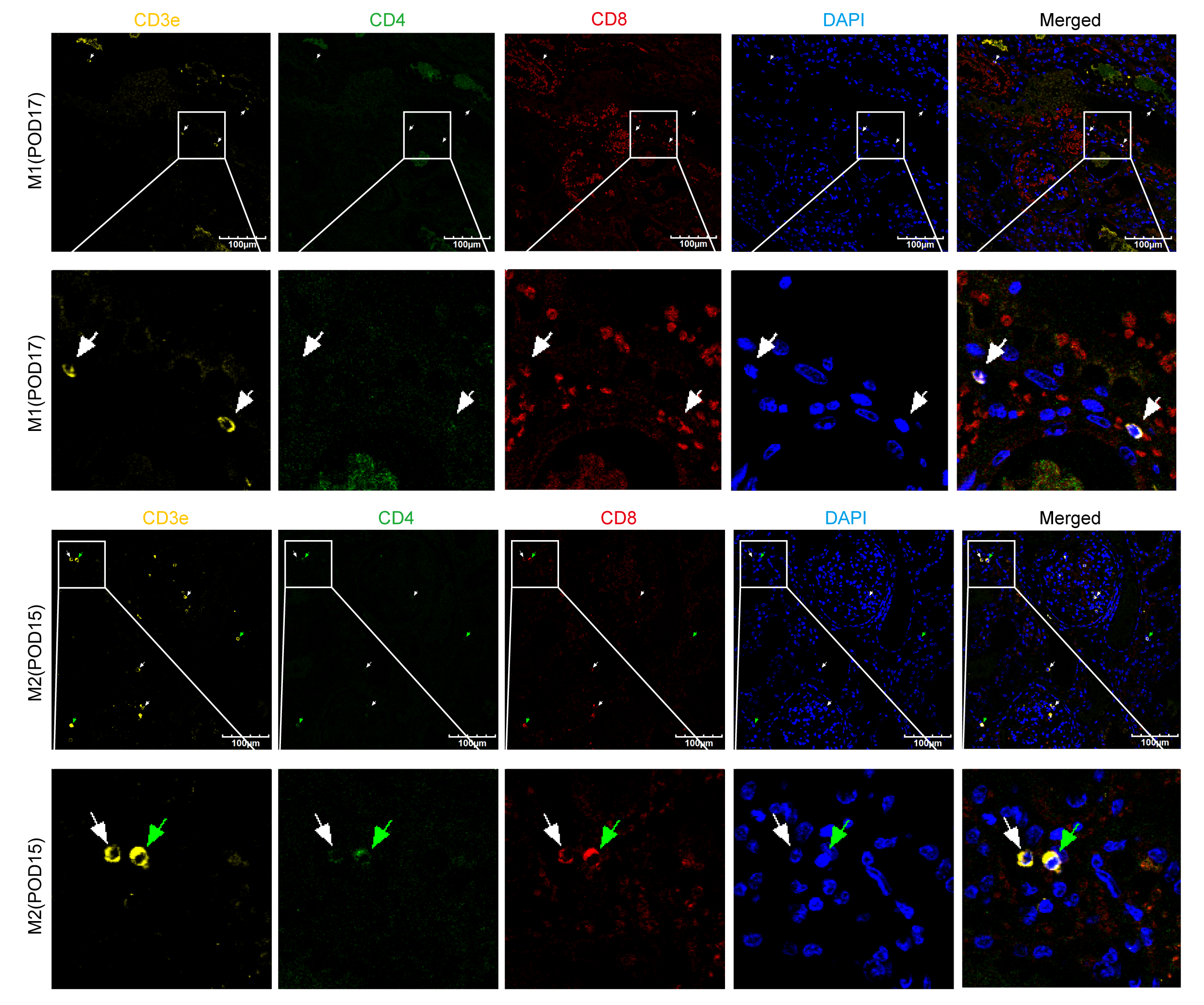

Supplement: Supplementary file 6 — Figure S6. T cell infiltration in porcine kidney xenograft confirmed by immunofluorescence (scale bar = 100 μm). [file CPR-58-e70028-s007.tif]
