# Supplementary material for: Production and Functional Verification of 8‐Gene (GGTA1, CMAH, β4GalNT2, hCD46, hCD55, hCD59, hTBM, hCD39)‐Edited Donor Pigs for Xenotransplantation
Source: Cell Prolif. 2025 Apr 6;58(9):e70028. doi: 10.1111/cpr.70028 (PMC12414638; doi:10.1111/cpr.70028)
Supplement: Supplementary file 7 — Table S1. Primers for genotyping, qPCR and ddPCR. [file CPR-58-e70028-s005.docx]

Table S1 Primers for genotyping, qPCR and ddPCR

| Primers | Sequence (5’-3’) | Size |
| --- | --- | --- |
| Genotyping | | |
| GGTA1-F | GGCAACATGGCAGGAAGGAA | 775bp |
| GGTA1-R | AGACGGCCCTGTCAGTTCAT |  |
| CMAH-F | AGCCTTAGAAGCCAGTGCAG | 904bp |
| CMAH-R | GCCCAAGCGAATCTACCTCA |  |
| β4GalNT2-F | TGCTCCATCGCAAGTGAC | 758bp |
| β4GalNT2-R | CCCGCAGTCAGGATTCAC |  |
| hCD55&hCD59-F | TCCAGCACCACCACAAATTGAC | 1124bp |
| hCD55&hCD59-R | CGGTGACCCGCTCGATGTG |  |
| hCD46-F | CTGATGAGACCCACAGAG | 100bp |
| hCD46-F | GCTCCACCATCTGCTTTC |  |
| hTBM&hCD39-F | GCTCGTGCATTCGGGCTTGC | 766bp |
| hTBM&hCD39-R | CCTGGCACCCTGGAAGTCAAAG |  |
| hTBM&hCD39-F | CCTTCCTCAATGCCAGTCAG | 2168bp |
| hTBM&hCD39-R | CCTGGCACCCTGGAAGTCAAAG |  |
| qPCR | | |
| hCD46-F | ccaggtgcaggatcacaact | 146bp |
| hCD46-R | gcaatttggagcggtaagc |  |
| hCD55-F | TCCAGCACCACCACAAATTGAC | 207bp |
| hCD55-R | GGTGGGACCTTGGAAGTTAGAG |  |
| hCD59-F | TCATAGCCTGCAGTGCTACAAC | 103bp |
| hCD59-R | CCCAGCTTTGGTAATGAGACAC |  |
| hTBM-F | CATCCTGGACGACGGTTTCA | 107bp |
| hTBM-R | CGCAGATGCACTCGAAGGTA |  |
| hCD39-F | GGTGCCTATGGCTGGATTA | 211bp |
| hCD39-R | CCTTGCCATAGAGGCGAAA |  |
| pGAPDH-F | AGGGCATCCTGGGCTACACT | 367bp |
| pGAPDH-R | TCCACCACCCTGTTGCTGTAG |  |
| hGAPDH-F | GAGTCAACGGATTTGGTCGT | 160bp |
| hGAPDH-R | TGGAAGATGGTGATGGGATT |  |
| ddPCR | | |
| hCD46-F | CGTGGTCTCTTCTGCCTATTT | 95bp |
| hCD46-R | AAGGAAACTGAGACGCTACTG |  |
| hCD46 Probe | FAM TCCAGTGAAAGAAGCCAAGATCAGTAAGC |  |
| hCD55-F | GGTGCCAACAAGGCTAAATTC | 99bp |
| hCD55-R | CCTGGACGGCACTCATATTC |  |
| hCD55 Probe | FAM TGCATCCCTCAAACAGCCTTATATCACT |  |
| hCD59-F | CTGCAAGAAGGACCTGTGTAA | 93bp |
| hCD59-R | AATGGAGTCACCAGCAGAAG |  |
| hCD59 Probe | FAM AATGGTGGGACATCCTTATCAGAGAAA |  |
| hTBM-F | ACGTGGATGACTGCATACTG | 100bp |
| hTBM-R | ACCAGGTCGTAGTTAGGGTAG |  |
| hTBM Probe | FAM TCAACACACAGGGTGGCTTCGAG |  |
| hCD39-F | GCTCTGCAATTTCGCCTCTAT | 106bp |
| hCD39-R | GAATGTCCTTGGCCAGTTTCT |  |
| hCD39 Probe | FAM CTTCTTGTGCTATGGGAAGGATCAGGC |  |
| hGAPDH-F | CCTAGGGCTGCTCACATATTC | 85bp |
| hGAPDH-R | CGCCCAATACGACCAAATCTA |  |
| hGAPDH Probe | HEX CTCATGCCTTCTTGCCTCTTGTCTCT |  |
| pGAPDH-F | ccgcgatctaatgttctctttc | 114bp |
| pGAPDH-R | ttcactccgaccttcaccat |  |
| pGAPDH Probe | HEX cagccgcgtccctgagacac |  |
