# Supplementary material for: Production and Functional Verification of 8‐Gene (GGTA1, CMAH, β4GalNT2, hCD46, hCD55, hCD59, hTBM, hCD39)‐Edited Donor Pigs for Xenotransplantation
Source: Cell Prolif. 2025 Apr 6;58(9):e70028. doi: 10.1111/cpr.70028 (PMC12414638; doi:10.1111/cpr.70028)
Supplement: Supplementary file 8 — Table S2. List of antibodies used for protein expression. [file CPR-58-e70028-s009.docx]

Table S2 A summary of antibodies used by immunofluorescence, immunohistochemistry and flow cytometry

|  | Primary antibody | | Secondary antibody | |
| --- | --- | --- | --- | --- |
|  | Catalog | Manufacturer | Catalog | Manufacturer |
| Immunofluorescence | | | | |
| GGTA1 | ALX-650-001F-MC05 | Enzo |  |  |
| CMAH(Neu5Gc) | 146903 | BioLegend | 550021 | Zenbio |
| B4GalNT2(DBA) | FL-1031 | Vector Labs |  |  |
| hCD46 | ab108307 | Abcam | GB21303 | Servicebio |
| hCD55 | ab230638 | Abcam | GB21303 | Servicebio |
| hCD55 | ab133684 | abcam | GB21303 | Servicebio |
| hCD59 | BF0017 | Affinity | GB21301 | Servicebio |
| hCD59 | ab9183 | abcam | GB21301 | Servicebio |
| hTBM(CD141) | sc-13164 | Santa Cruz | GB21301 | Servicebio |
| hCD39 | ab223842 | Abcam | GB21303 | Servicebio |
| anti-CD3e | 85061T | CST | GB21303 | Servicebio |
| anti-CD4 | A22773 | ABClone |  |  |
| anti-CD8 | A23346 | ABClone |  |  |
| IgG | 62-8411 | Invitrogen |  |  |
| IgM | A18842 | Invitrogen |  |  |
| C3c | RAB-0027 | MXB Biotech. | GB21303 | Servicebio |
| C4d | RMA-0857 | MXB Biotech. | GB21303 | Servicebio |
| C5b-C9 | MA5-28502 | Invitrogen | GB21301 | Servicebio |
| Immunohistochemistry | | | | |
| CD57 | 14-0577-82 | Invitrogen | PV-9000 | OriGene |
| CD68 | 76437S | Cell Signaling | PV-9000 | OriGene |
| Flow cytometry | | | | |
| GGTA1 | ALX-650-001F-MC05 | Enzo |  |  |
| CMAH(Neu5Gc) | 146903 | BioLegend | 550021 | Zenbio |
| B4GalNT2(DBA) | FL-1031 | Vector Labs |  |  |
| hCD46 | 315304 | Biolegend |  |  |
| hCD55 | 555694 | BD |  |  |
| hCD59 | MA1-19463 | Invitrogen |  |  |
| hTBM | 564123 | BD |  |  |
| hCD39 | 560239 | BD |  |  |
