# Supplementary material for: Production and Functional Verification of 8‐Gene (GGTA1, CMAH, β4GalNT2, hCD46, hCD55, hCD59, hTBM, hCD39)‐Edited Donor Pigs for Xenotransplantation
Source: Cell Prolif. 2025 Apr 6;58(9):e70028. doi: 10.1111/cpr.70028 (PMC12414638; doi:10.1111/cpr.70028)
Supplement: Supplementary file 11 — Table S5. Annotation information of transgenic insertion location. [file CPR-58-e70028-s011.docx]

Table S5 Annotation information of transgenic insertion location

| No. | Chr. | Left location of insertion | Right location of insertion | Annotation information | |
| --- | --- | --- | --- | --- | --- |
| 1 | chr13 | 3515829 | 3515830 | Intron region of RFTN1 gene | Exon6: 3514487-3514690 |
|  |  |  |  |  | Intron: 3514691-3524215 |
|  |  |  |  |  | Exon5: 3524216-3524567 |
| 2 | chr16 | 6136551 | 6136552 | Intron region of MYO10 gene | Exon2: 6094164-6094262 |
|  |  |  |  |  | Intron: 6094263-6144945 |
|  |  |  |  |  | Exon1: 6144946-6145485 |
| 3 | chr16 | 20932562 | 20932563 | Intergenic region between C1QTNF3 and LOC110257267 genes | C1QTNF3: 20032973-20059905 |
|  |  |  |  |  | LOC110257267: 20106984-20145241 |
|  |  |  |  |  | Intergenic region: 20059906-20106983 |
